# Investigation of the gastric digestion behavior of commercial infant formulae using an *in vitro* dynamic infant digestion model

**DOI:** 10.3389/fnut.2024.1507093

**Published:** 2024-12-05

**Authors:** Faith Bernadette Descallar, Debashree Roy, Xin Wang, Peter Zhu, Aiqian Ye, Yichao Liang, Shikha Pundir, Harjinder Singh, Alejandra Acevedo-Fani

**Affiliations:** ^1^Riddet Institute, Massey University, Palmerston North, New Zealand; ^2^Fonterra Research and Development Centre, Palmerston North, New Zealand

**Keywords:** infant formula, *in vitro* dynamic digestion, milk proteins, biopolymers, gastric emptying

## Abstract

The gastric digestion behavior of different commercial Stage 1 infant formulae (for 0–6 months) with different formulation backgrounds was investigated using an *in vitro* dynamic infant human gastric simulator (iHGS). The microstructural arrangements of the protein and lipid, colloidal stability and protein hydrolysis during digestion were elucidated. During gastric digestion, casein-dominant formulations showed a higher extent of aggregation due to their high proportion of casein micelles that underwent coagulation upon acidification and via the action of pepsin. The extensive protein coagulation/curd formation in casein-dominant infant formulae slowed the rate of protein hydrolysis and resulted in the retention of caseins in the iHGS for longer times. Confocal micrographs showed that oil droplets were entrapped in the curd particles of casein-dominant infant formulae, which consequently slowed the gastric emptying of lipids. Conversely, whey-dominant formulations showed a lower degree of protein aggregation that resulted in faster protein hydrolysis and rapid protein and lipid emptying from the iHGS. It was also revealed that whey-dominant infant formulae in the presence of biopolymers increased the viscosity of gastric chyme and induced the flocculation of oil droplets. This altered the rate of protein hydrolysis and emptying of lipids. Correlation analyses depicted the overall kinetics of gastric emptying of macronutrients during digestion and comprised two stages: (i) driven by the continuous stomach emptying and (ii) influenced by aggregation and coalescence indices. The present study highlights the similarities and differences in the digestion behaviors of commercial infant formulae based on important ingredients such as types of proteins and biopolymers, regardless of the formulation or processing histories.

## 1 Introduction

Breast milk is the best source of nutrition for the neonate. Typically, exclusive breastfeeding is recommended within the first 6 months of life, continuing with a complementary feeding with breast milk and weaning food until 2 years-old or beyond (1). Human breast milk contains a perfect balance of macro-and micronutrients to fulfill the neonate’s growth requirements such as water, carbohydrates (7%), protein (~1%), and fat (~3.8%) (2, 3).

When breastfeeding is not sufficient or difficult, using breast milk substitutes such as infant formula (IF) is the next option to cover neonates’ nutritional requirements. Whole milk, skim milk, and whey protein powders from cow, goat or sheep are used as protein sources in infant formulae in different proportions to meet specific nutritional needs. For instance, Stage 1 infant formulae designed for 0–6 month old infants tend to be whey-dominant formulations with a typical whey to casein ratio of 60:40. On the other hand, casein-dominant formulae are more common in Stage 2 products as they can be slower to digest for the newborn, and are commonly used to provide satiation of infants for longer times (4).

In some cases, however, formula-fed neonates suffer from digestive issues because of their immature gastrointestinal tract; compared with adults, their enzymatic activity and gastric motility are lower (5). Some of the most common gastrointestinal issues include gastroesophageal reflux, colic, constipation, and diarrhea (6). To alleviate some of these problems, infant formula manufacturers offer special formulations claiming certain digestive benefits. A commonly used intervention is to thicken or increase the viscosity of infant milk, which prevents the retrograde flow of gastric content to the esophagus (7–9).

Choosing the correct formulation is crucial for all infants, but it is particularly more important during the first 6 months as infants require precise nutrients and sufficient immune support that are vital for their growth and development. For infants aged 0–6 months, digestion of relevant nutrients from milk starts in the stomach since the transition time of liquid milk in the oral phase (mouth, pharynx, and esophagus) is very short (~10–15 s) (10, 11). Thus, this study focused on exploring the digestion behavior of Stage 1 IFs in the gastric environment.

It is well established that the kinetics of digestion and physiological properties of milk are influenced by food composition, processing treatments, and physical and structural properties of macronutrients (12–14). Over the years, gastrointestinal studies on infant formulae have received much attention due to a need to better understand the digestion process and elucidate the differences and similarities between formulae and human breastmilk. Bourlieu et al. (15) reported on the lipolysis and structural changes during *in vitro* digestion comparing human milk and five commercial infant formulae. Yuan et al. (16) compared four commercial infant formulae powders and human milk exploring the effect of interfacial materials and size on the lipid droplets on *in vitro* digestion. Chauvet et al. (17) formulated four model infant formula powders with different protein ingredients and studied their impacts on digestion using the DIDGI^®^ bi-compartmental system (18).

These published reports used a static or semi-dynamic system which limits their physiological relevance to create the conditions driving the dynamic mixing and disintegration of the gastric contents. Recently, Song et al. (19) studied the dynamic *in vitro* gastric digestion behavior of infant formulae using an infant human gastric simulator (iHGS) model; however, this work only focused on three commercial formulae from different sources. This dynamic iHGS model is more sophisticated than the static models as it simulates the stomach contraction patterns, gradual secretion of gastric fluids, body temperature, and gastric emptying (20–23). Although several studies have been published on commercial infant formulae, no systematic study has compared the digestion properties of a range of different infant formulations from different commercial suppliers (with varying processing history) using an *in vitro* dynamic model.

Thus, the aim of this study was to investigate the gastric digestion behavior of ten different Stage 1 commercial infant formulae with variable compositions (e.g., whey to casein ratios, presence of biopolymers) and relate how specific ingredients impact the kinetics of macronutrient delivery to the small intestinal phase using an *in vitro* dynamic gastric simulator model (iHGS). The microstructural rearrangement of protein and lipids as well as the degree of intragastric stability were also elucidated and new correlation models were developed to describe these changes.

## 2 Materials and methods

### 2.1 Materials and sample preparation

Market-available infant formulae specifically labeled for 0–6 months infants were purchased from supermarkets in two different countries (New Zealand and China) and the proximate protein compositions according to the product label are shown in Table 1. A complete list of ingredients based on the product labels is shown in Supplementary Table S1. Pepsin from porcine gastric mucosa (P7012; 2,500 units/mg solid) was purchased from Sigma-Aldrich Corporation (St. Louis, MO, United States). Gastric lipase from *Aspergillus niger* fungus (A “Amano” 12, LU0451409K, 1,200 units/mg) was purchased from Amano Enzymes Incorporation (2–7, 1-Chome, Nishiki, Naka-Ku, Nagoya 460-8630, Japan). Water was purified by treatment with a Milli-Q apparatus (Millipore Corporation, Bedford, MA, United States) and was used for all experiments. All chemicals used were of analytical grade and were purchased from either Sigma Chemical Company Ltd. (St. Louis, MO, United States) or BDH Chemicals (BDH Ltd., Poole, United Kingdom) unless otherwise specified.

**Table 1:** Infant formulae product information on concentrations and source of main ingredients.

Sample	Protein (%)	Fat (%)	Whey:casein	Main milk ingredients
Casein IF 1	1.70	3.94	20:80	Cow milk solids
Casein IF 2	1.70	4.43	40:60	Goat milk
Casein IF 3	1.70	4.22	40:60	Sheep milk solids; sodium caseinate
Whey IF 1	1.70	3.35	60:40	Cow milk solids (demineralized whey powder, skim milk, lactose, whey protein concentrate)
Whey IF 2	1.70	4.18	60:40	Natural A2 protein milk (cow); A2 β-casein is ~34% of total casein
Whey IF 3	1.70	3.92	60:40	Cow milk solids (lactose, demineralized whey powder, whole milk, whey protein concentrate, skim milk); A2 β-casein is ~32% of total casein
Whey IF 4	1.70	3.64	60:40	Raw bovine milk, demineralized whey powder, casein phosphopeptide
Whey IF 5	1.70	3.79	66:34	Raw bovine milk, whey protein isolate, demineralized whey protein powder, whey protein concentrate, casein phosphopeptide
Whey IF 6	1.70	3.62	65:35	Cow milk solids, sodium caseinate, whey protein, thickener (carob bean gum)
Whey IF 7	1.70	3.94	100:0	Hydrolysed whey protein, starch

### 2.2 *In vitro* dynamic gastric digestion in an infant human gastric digestion

Each infant formula was reconstituted to a fixed 1.7% protein content (this resulted in differing fat contents of 3.4–4.6%) by completely dissolving the infant formula powder in deionized water at 50°C for 30 min. Gastric digestion of infant formulae was carried out using an iHGS adapted from previous studies (20–23). A schematic representation of the iHGS was previously reported by Ye et al. (22). The *in vitro* dynamic gastric digestion protocol in iHGS was developed considering the key parameters and most suitable conditions to mimic digestion of infants from 0 to 6 months (24). The simulated gastric fluid (SGF) was prepared as described by Minekus et al. (25) with slight modifications. The composition of the SGF and the parameters for the *in vitro* gastric digestion were based on our extensive review (24) and are summarized in Table 2.

**Table 2:** Parameters for the infant *in vitro* dynamic gastric digestion.

Component/condition	Constituent/parameter	Concentration/amount
Simulated gastric fluids (SGF)	KCl	13.2 mM
	NaCl	94 mM
	pH	2.0
Enzyme solution	Pepsin	651 U/mL SGF
	Lipase	60 U/mL SGF
Basal SGF	Fasted SGF without enzyme (pH 3.0)	10 g
Flow rate (1)	1.25× concentrated SGF (without pepsin and lipase)	0.4 mL/min
Flow rate (2)	Enzyme solution (pepsin and lipase)	0.1 mL/min
Infant formulae	Ingested amount	100 g
Gastric emptying	22 g gastric content per 20 min	
Gastric condition	37°C	
Gastric contraction	3 times per min	
Digestion time	Stopped at 15,40,80,120, 160 min for analysis	

Ten grams of basal SGF (pH 3.0) was added to 100 mL of reconstituted infant formula prior to digestion in the iHGS (26, 27). To simulate infant gastric secretion, the 1.25× concentrated SGF and the enzyme (pepsin and lipase) solution were pumped into the iHGS separately at flow rates of 0.4 and 0.1 mL/min, respectively (28, 29). To simulate infant gastric emptying, 22 mL of digesta samples were removed from the bottom of the iHGS at 20 min intervals. A mesh with a pore size diameter of 1 mm was placed inside the bottom of the iHGS to simulate gastric sieving (30). Experiments were terminated at specific digestion time point (15, 40, 80, 120, and 160 min), and samples were collected.

To analyze the gastric digestion kinetics of IF, two types of samples were collected: the gastric chyme (contents inside the stomach chamber) and the emptied digesta fraction from the bottom of the stomach (representing the fraction that moves to the small intestine). The gastric chyme and emptied digesta samples were collected and measured directly. To stop the enzymatic digestion, the pH of the gastric chyme and the digesta samples was raised immediately to 7.5 using 10 M NaOH.

### 2.3 pH measurement

The pH of the infant formulae during gastric digestion was measured using a benchtop pH meter (PL-700PV, Interlab, Wellington, NZ). The initial pH (0 min) was the measured pH of the reconstituted infant formula prior to digestion. With continuous injection of SGF and gastric emptying, the change of the pH inside the iHGS was assumed to be the same that of the emptied digesta because it was difficult to access into the stomach due to contractions of the latex stomach bag.

### 2.4 Particle size measurements

The mean particle size and particle size distributions of the initial infant formula, gastric chyme and emptied digesta were measured using a laser-light diffraction unit (Mastersizer 2000; Malvern Instruments, Malvern, Worcestershire, United Kingdom). The mean particle size of the samples particles was characterized by the volume-weighted average diameter [d_4,3_ (= *Σ* n_i_d_i_^4^/n_i_d_i_^3^), where n_i_ is the number of particles with diameter d_i_]. The refractive index of the samples was set at 1.457 (with an absorbance value of 0.001) and that of water was set at 1.33. A volume of initial infant formula, emptied digesta, and chyme were added to the dispersion unit until a laser obscuration range of 8 to 15% had been reached.

Mean particle diameters were calculated as the average of triplicate measurements on individual samples. The samples were also dispersed in a mixed solution of SDS (1%, w/w) and EDTA (50 mM) before measurement of particle size distribution. Briefly, approximately 500 μL of sample were diluted into 5 mL of SDS-EDTA buffer to disrupt the casein micelles and protein coagulate.

The degree of aggregation was determined from aggregation index (AI) as a normalized value of intragastric stability in the infant gastric environment. It was calculated using Equation 1 where d_4,3_(CH) is the volume-weighted mean diameter (d_4,3_) of the gastric chyme at a specific digestion time, and the d_4,3_(milk,t_0_) is the d_4,3_ value of the undigested formulation (31). An increase in AI denotes the level of aggregation that occurs during digestion because of flocculation and/or coalescence.


(1) $$\mathrm{AI}=\frac{d_{4,3}\left(CH\right)-d_{4,3}\left(\mathrm{milk},t_{0}\right)}{d_{4,3}\left(\mathrm{milk},t_{0}\;\right)}$$

Similarly, we determined the degree of lipid droplets destabilization by calculating the Coalescence Index (CI) that is derived from the d_4,3_ of the gastric chyme dispersed in SDS + EDTA buffer. This index indicates the instability of interfacial layer during the digestion process. The higher the CI, the lower the stability of interfacial layer of oil droplets during digestion. The equation of CI is shown in Equation 2 where d_4,3_(CH in buffer) and d_4,3_(milk in buffer, t_0_) are the mean particle size of the gastric chyme and undigested milk dispersed in SDS + EDTA buffer, respectively.


(2) $$CI=\frac{d_{4,3}\left(CH\;\mathrm{in}\;\mathrm{buffer}\right)-d_{4,3}\left(\mathrm{milk}\;\mathrm{in}\;\mathrm{buffer},t_{0}\right)}{d_{4,3}\left(\mathrm{milk}\;\mathrm{in}\;\mathrm{buffer},t_{0}\;\right)}$$

### 2.5 Viscosity measurements

The viscosity of each of the formulae was measured using a TA HR 20 rheometer (TA Instruments Corporate, New Castle, DE 19720, United States). The temperature of sample was set at 37°C. Logarithmic sweep shear rate was measured from 1 to 100 1/s using double wall concentric cylinder (19280) geometry.

### 2.6 Confocal laser scanning microscopy

The microstructure of the undigested and digested infant formulae was investigated using confocal laser scanning microscopy (CLSM) with a Leica SP5 DM6000B confocal laser scanning microscope (Leica Microsystems, Heidelberg, Germany). The samples were stained and observed immediately after collection. The fluorescent dye Nile Red (0.1% in acetone, w/v) was used to stain the oil phase (argon laser with excitation at a wavelength of 488 nm). Fast Green (1.0%, w/v) was used to stain protein (He–Ne laser with excitation at 633 nm). A 200 μL aliquot of the undigested and digested infant formula was transferred into an Eppendorf tube and gently mixed with 5 μL of 1.0% (w/v) Fast Green and 10 μL of 0.1% (w/v) Nile Red. Observations were made at least 5 min after diffusion of the dyes into the samples. The samples were placed on concave confocal microscope slides (Sail; Sailing Medical-Lab Industries Co. Ltd., Suzhou, China) covered with coverslips and observed with a 63× oil immersion lens (Leica HCX PL APO lambda blue 63.0 × 1.40 OIL UV, Wetzlar, Germany). Micrographs were stored with 1,024 × 1,024-pixel resolution and analyzed using ImageJ.

### 2.7 Chemical analyses

Chemical analyses, which included dry matter, total nitrogen, and lipid contents, were performed on the undigested formulae, gastric chyme, and emptied digesta samples. The dry matter was determined by placing the samples in dry aluminum containers and dried in an air oven at 105°C until constant weight was attained. The total nitrogen contents were determined using the Dumas method (AOAC 968.06) (32) and crude protein was calculated using a conversion factor of 6.38 for milk. Lipid contents were measured using the Mojonnier method (AOAC 989.05) (33).

### 2.8 Protein hydrolysis

The extent of hydrolysis of protein by pepsin was determined by analyzing the protein composition of the samples as a function of the digestion time, using sodium dodecyl sulfate-polyacrylamide gel electrophoresis (SDS-PAGE) under reducing conditions. One hundred microlitres of each sample was mixed with an electrophoresis sample buffer to obtain an equal protein content of 0.2% (w/v), removing the effect of dilution by simulated gastric secretion (34). The sample buffer contained 13% (v/v) 0.5 M Tris–HCl buffer, pH 6.8, 10% (v/v) glycerol, 2% (w/v) SDS, 0.04% (w/v) bromophenol blue and *β*-mercaptoethanol (19:1, v/v). The samples were kept at ambient temperature and an 8 μL aliquot of the solution was loaded onto a precast gel Criterion™ Tris-Tricine Gel (16.5%, 18 well, 30 μL #3450064) from Bio-Rad Laboratories (Hercules, CA, United States). Applied voltage was 125 V and conducted for approximately 80 min. The gel was stained for 40 min under gentle shaking using a Coomassie Brilliant Blue R staining solution [0.003% (w/v) in 10% (v/v) acetic acid (BDH) and 20% (v/v) isopropanol (Merck, Darmstadt, Germany)]. The gel was destained with a destaining solution of 10% (v/v) acetic acid and 10% (v/v) isopropanol and scanned using a Molecular Imager Gel Doc XR system (Bio-Rad Laboratories). Precision Plus Protein™ Dual Xtra Prestained Protein Standards (Bio-Rad Laboratories) were loaded for estimations of molecular mass. The protein composition from the SDS-PAGE gel was quantified by densitometry using Image Lab™ software version 5.2 (Bio-Rad Laboratories).

### 2.9 Statistical analysis

The pH results and standard deviations reported are from the pH of the emptied digesta from 2–6 digestion trials. One-way analysis of variance (ANOVA) model was performed to test the significance in variation using MS Excel. *Post hoc* tests at a significant value of *p* < 0.05 were also conducted using Tukey’s range test when the *F* value was significant (*p* < 0.05).

Gastric retentions were calculated from the volume of gastric chyme remaining in the stomach and total volume of the food material and gastric juice at given digestion time points. A modified power exponential model based on Elashoff’s model was fitted into the gastric retention data as expressed in Equation 3 (35–37):


(3) $$y\left(t\right)=\alpha_{0}\exp-{\left(\kappa t\right)}^{\beta }$$

where y(t) is the fractional meal retention at time t (min), α_0_ is the proportion remaining at t = 0 (100% for the relative retention), *κ* is the slope of the curve or the gastric emptying rate per minute, *β* is the index of the shape of the curve.

The results reported on the correlation of protein and lipid emptied were fitted using linear regression and the goodness of fitting was determined from the coefficient of determination (R^2^).

## 3 Results and discussion

### 3.1 Characteristics of reconstituted infant formulae

**Figure 1:**
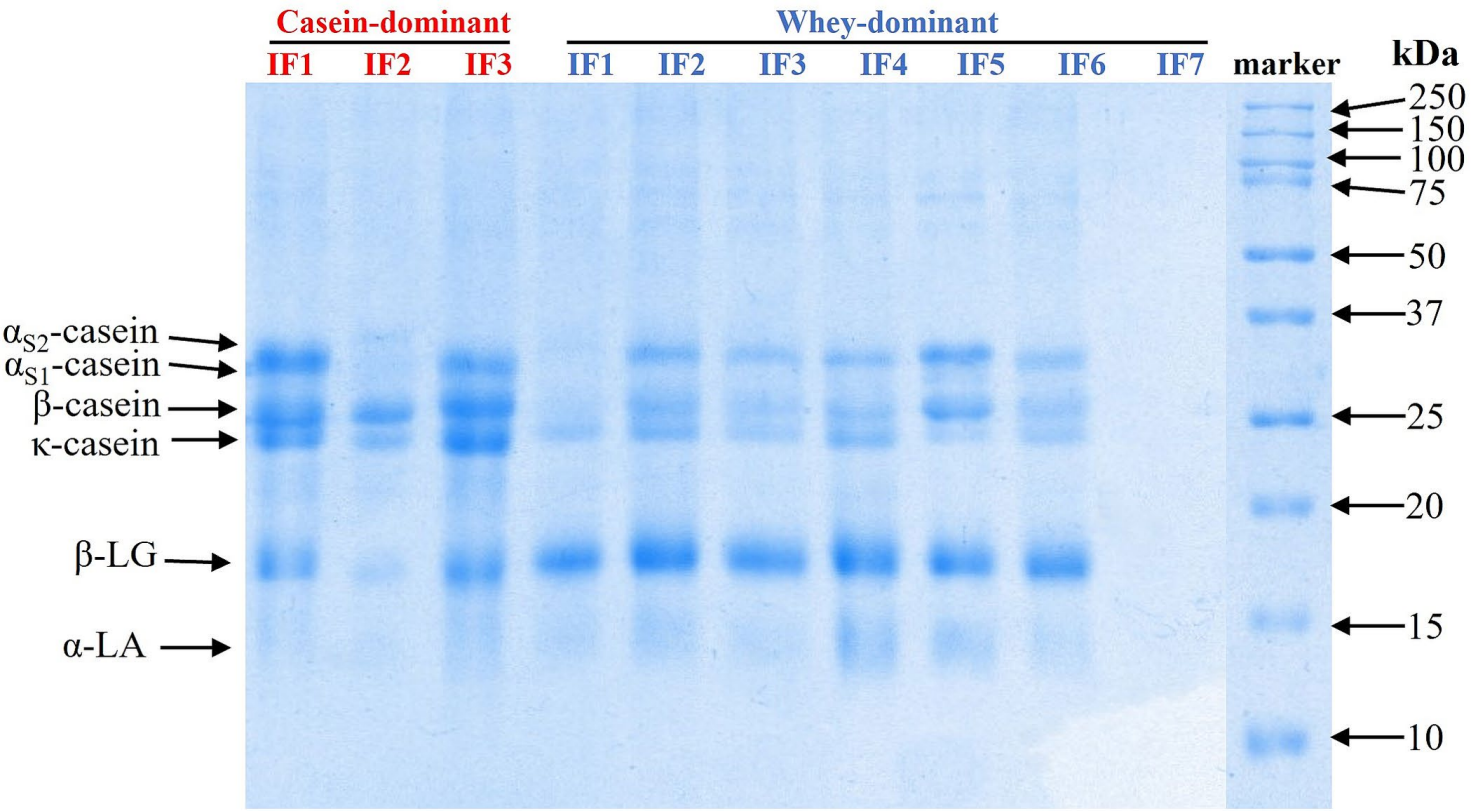
SDS-PAGE patterns under reducing conditions of the undigested infant formulae.

The protein composition of the infant formulae was examined using SDS-PAGE (Figure 1). As expected, casein-dominant IFs showed higher proportions of caseins, while whey-dominant IFs (except whey IF 7) showed higher fractions of *α*-lactalbumin (α-LA) and *β*-lactoglobulin (β-LG). Whey IF 7 showed no visible bands of intact milk proteins, confirming that the formulation was made with hydrolyzed whey proteins. These results agree with the manufacturer’s claims of casein-to-whey ratios.

**Figure 2:**
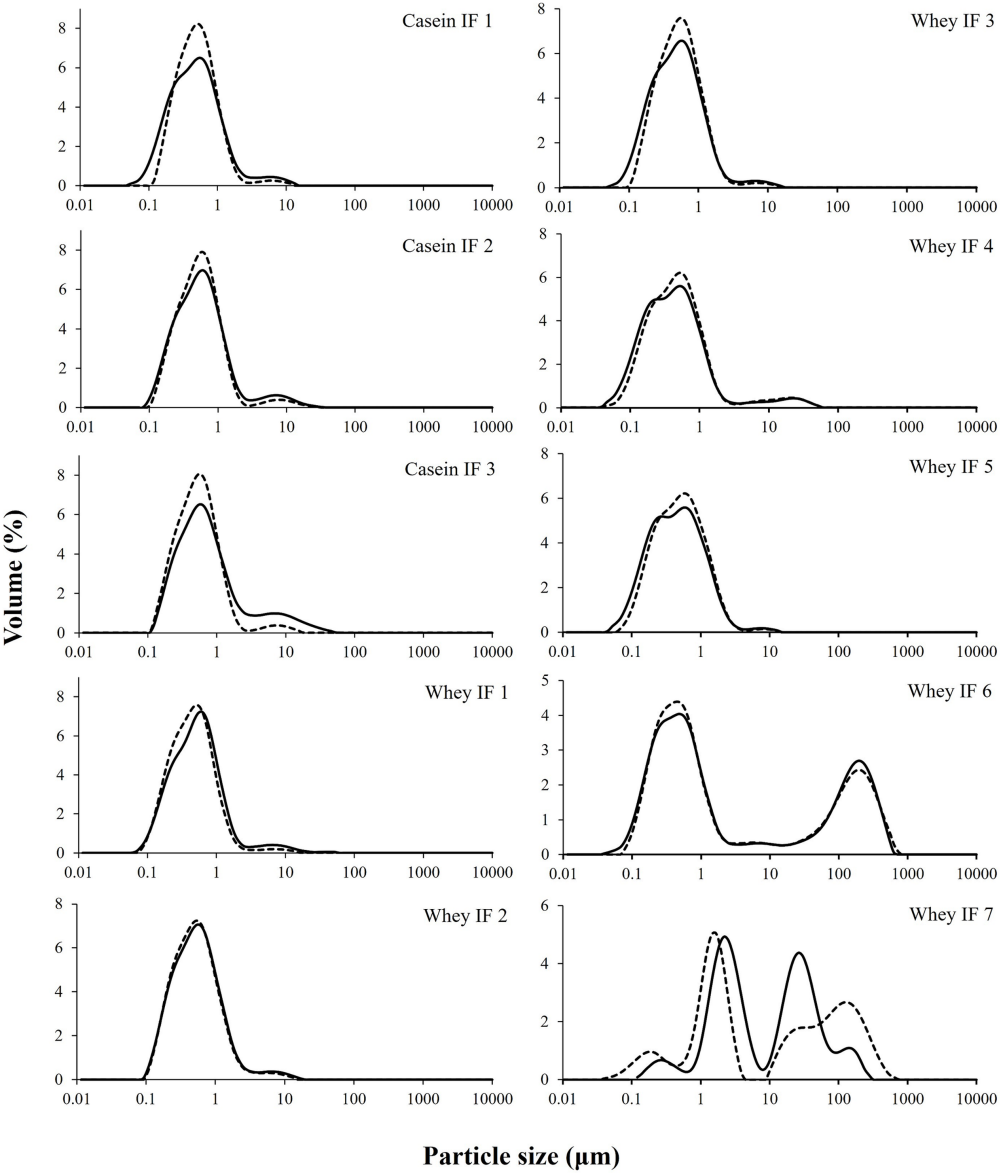
Particle size distributions of infant formulae dispersed in water (solid line) or in SDS + EDTA buffer (broken line).

The volume-weighted mean diameter (d_4,3_) and particle size distributions of the undigested reconstituted infant formulae are shown in Table 3 and Figure 2, respectively. Most infant formulae showed a bimodal distribution, characterized by a major peak located between 0.04 and 5 μm, except for whey IF 7 and IF 6. Whey IF 6 showed two distinct major peaks: one around 0.03–5 μm and another around 80–1,000 μm. In addition, whey IF 7 showed a multimodal distribution with particles population ranging between 0.04 and 725 μm. This distribution in whey IFs 6 and 7 was possibly due to the presence of thickeners (carob bean gum and starch) in the formulation. After mixing the reconstituted formula with SDS + EDTA buffer, the d_4,3_ values of whey IFs 6 and 7 remained large, which suggests that the large particles were due to the presence of biopolymers in the formulation. Biopolymers mentioned in this work pertain to the carob bean gum and starch present in the sample formulations of whey IF 6 and whey IF 7, respectively.

**Table 3:** Volume-weighted mean diameter (d_4,3_) of infant formulae dispersed in water or in SDS + EDTA buffer solution.

Sample	d_4,3_ (μm)	
	Water	SDS + EDTA buffer
Casein IF 1	0.78 ± 0.07	0.66 ± 0.03
Casein IF 2	1.24 ± 0.19	0.96 ± 0.12
Casein IF 3	1.80 ± 0.17	0.65 ± 0.14
Whey IF 1	0.98 ± 0.04	0.61 ± 0.03
Whey IF 2	0.86 ± 0.07	0.75 ± 0.06
Whey IF 3	0.69 ± 0.03	0.65 ± 0.03
Whey IF 4	1.61 ± 0.07	1.75 ± 0.11
Whey IF 5	0.65 ± 0.03	0.68 ± 0.02
Whey IF 6	59.65 ± 11.39	54.62 ± 14.69
Whey IF 7	24.74 ± 4.82	66.65 ± 25.36

Conversely, the d_4,3_ of other infant formulae slightly decreased after dispersion in the SDS + EDTA buffer solution, which could be due to a dissociation of flocculated droplets. Casein-dominant IFs showed a steep decrease in the d_4,3_ after dispersion in the SDS + EDTA buffer, which suggests greater droplet flocculation in the casein-dominated IFs compared with whey-dominated IFs. This is related to the high proportion of casein micelles in these formulations which tend to induce bridging flocculation between the oil droplets during emulsification (22).

**Figure 3:**
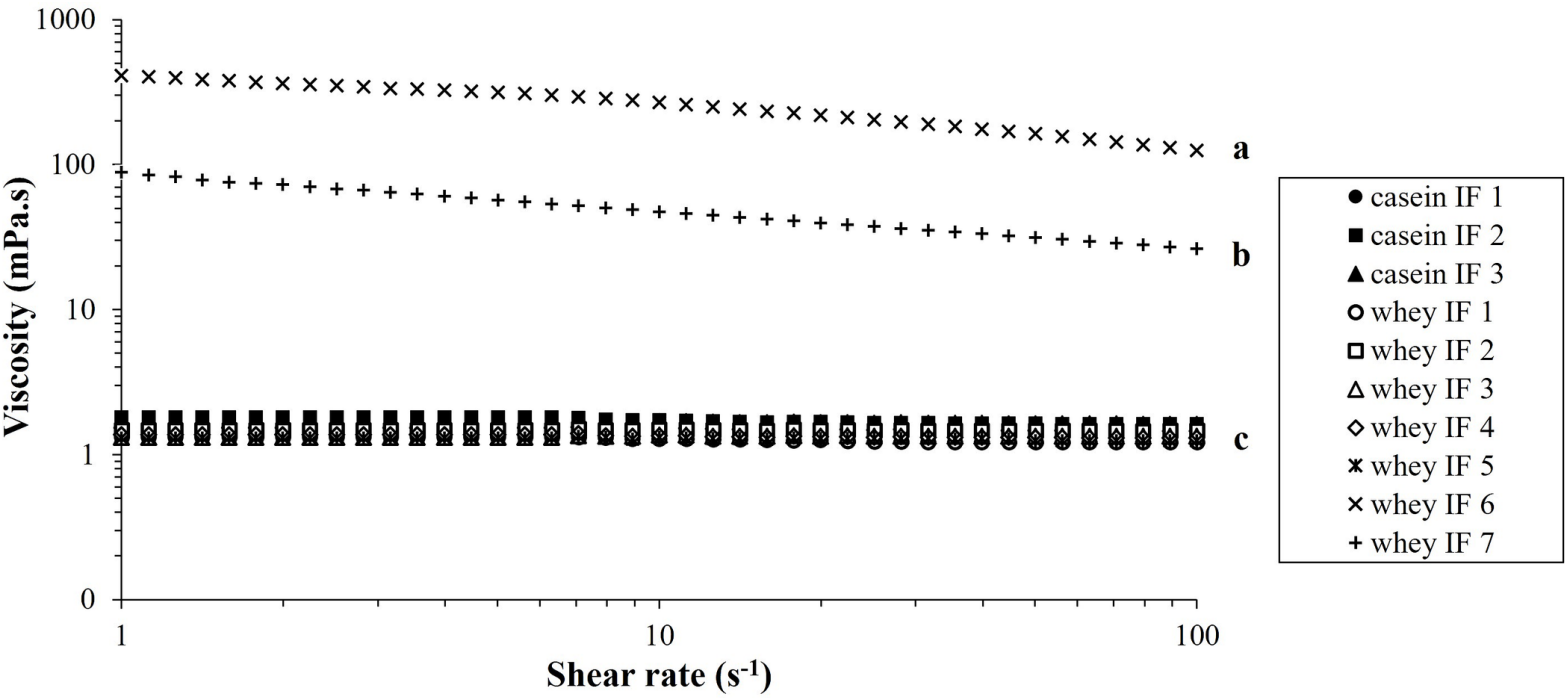
Viscosity versus shear rate profiles of the reconstituted infant formulae at 37°C. Different lowercase letters (a, b, c) indicate significant difference (*p* < 0.05) in the viscosity of the different infant formulae prior digestion.

The shear viscosity of reconstituted infant formulae is shown in Figure 3. All the infant formulae (except whey IFs 6 and 7) showed a linear viscosity (approximately 1.5 mPa.s) with increased shear rate suggesting a Newtonian behavior. Viscosity profiles of whey IFs 6 and 7 showed a non-Newtonian shear-thinning behavior which is due to the presence of thickeners in these formulations. Polysaccharides, such as starch and carob bean gum, are prone to conformational changes, interactions, and molecular alignment as the shear in the flow field is increased (38, 39). Interestingly, the shear viscosity of whey IF 6 was also higher than of whey IF 7. This could be attributed to the fact that carob bean gum (added to whey IF 6) has higher gelling ability compared with starch (added to whey IF 7) (40, 41).

Due to the difference in the physical properties of the IFs, it is expected that the gastric digestion behaviors would be influenced by their initial viscosity in addition to the different protein composition and particle size distributions. From here, samples are categorized into three groups namely: casein-dominant IFs (casein IFs 1, 2 and 3), whey-dominant IFs (whey IFs 1, 2, 3, 4, and 5), and biopolymer-stabilized whey-dominant IFs (whey IFs 6 and 7).

### 3.2 *In vitro* dynamic gastric digestion

#### 3.2.1 Gastric pH profile

**Figure 4:**
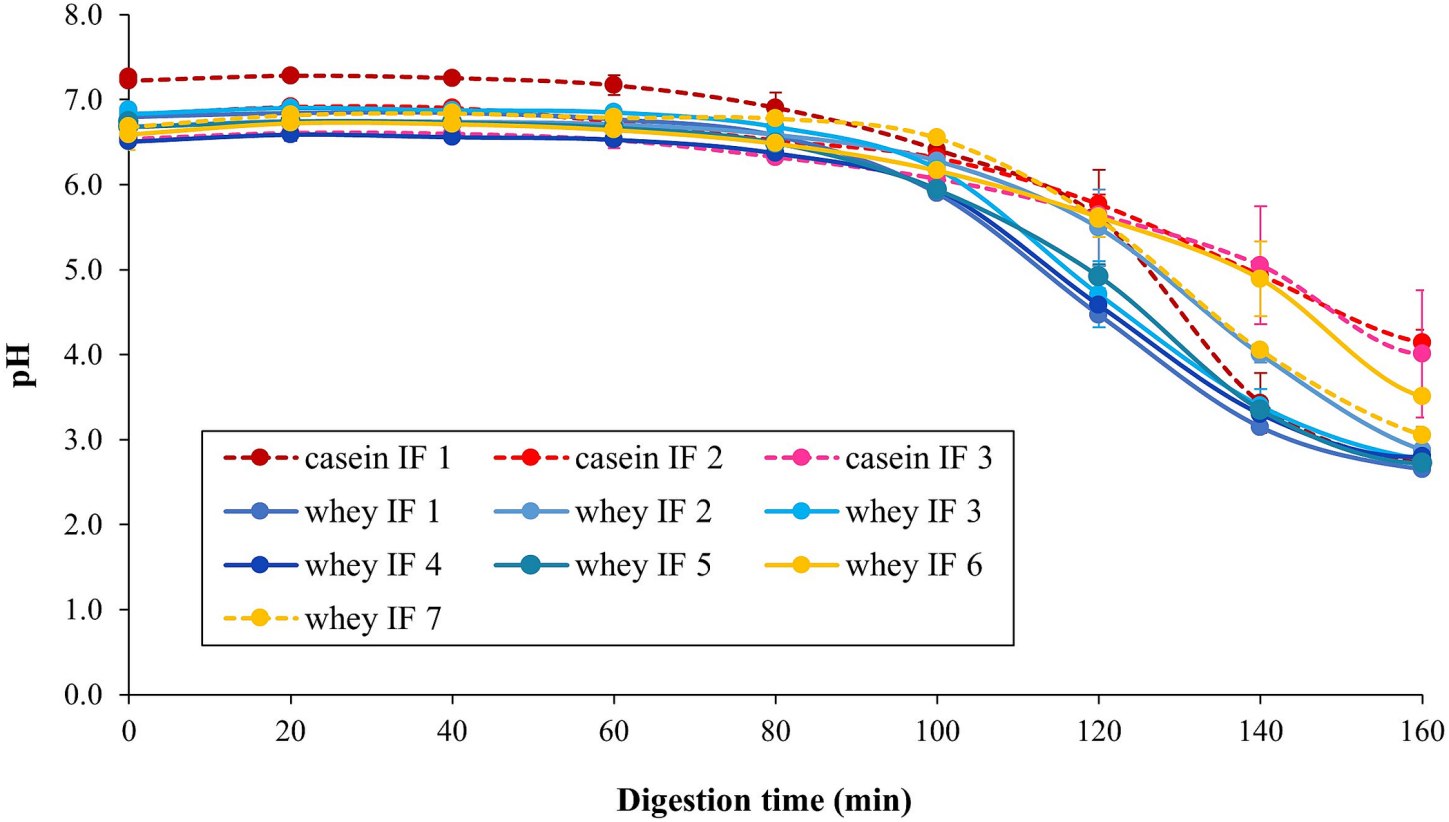
Change in pH of the infant formulae during gastric digestion in the iHGS. Error bars correspond to the standard deviation from 2 to 6 trials. Different lowercase letters (a, b, c) indicate significant difference (*p* < 0.05) in the pH of the different infant formulae at specific digestion time points.

The changes in pH values of the infant formulae during simulated gastric digestion are shown in Figure 4. With the continuous addition of SGF and enzymes (pepsin and lipase) to the iHGS, the pH of all infant formulae decreased as digestion time increased (42). There was no significant difference in the pH versus time profile among all formulae, except for casein IF 1 at the earlier digestion times (before 100 min). After 100 min, the pH of casein IF 1 drastically decreased to ~3.0–4.0. In addition, casein IF 2, casein IF 3, and whey IF 6 showed a slightly higher pH than the other formulations at 160 min, which suggests their higher buffering capacity. The higher buffering capacity of casein-dominant IFs was possibly due to presence of casein micelles with associated micellar calcium phosphate and a higher protein retention during digestion. The slightly higher final pH of whey IF 6 could be related to the presence of a thickener (carob bean gum) in the formulation. The carob bean gum induced a significantly higher viscosity in the formulation compared with all other IFs (Figure 3) and this could have slowed down the diffusion of SGF (43).

#### 3.2.2 Apparent visual changes during digestion

**Figure 5:**
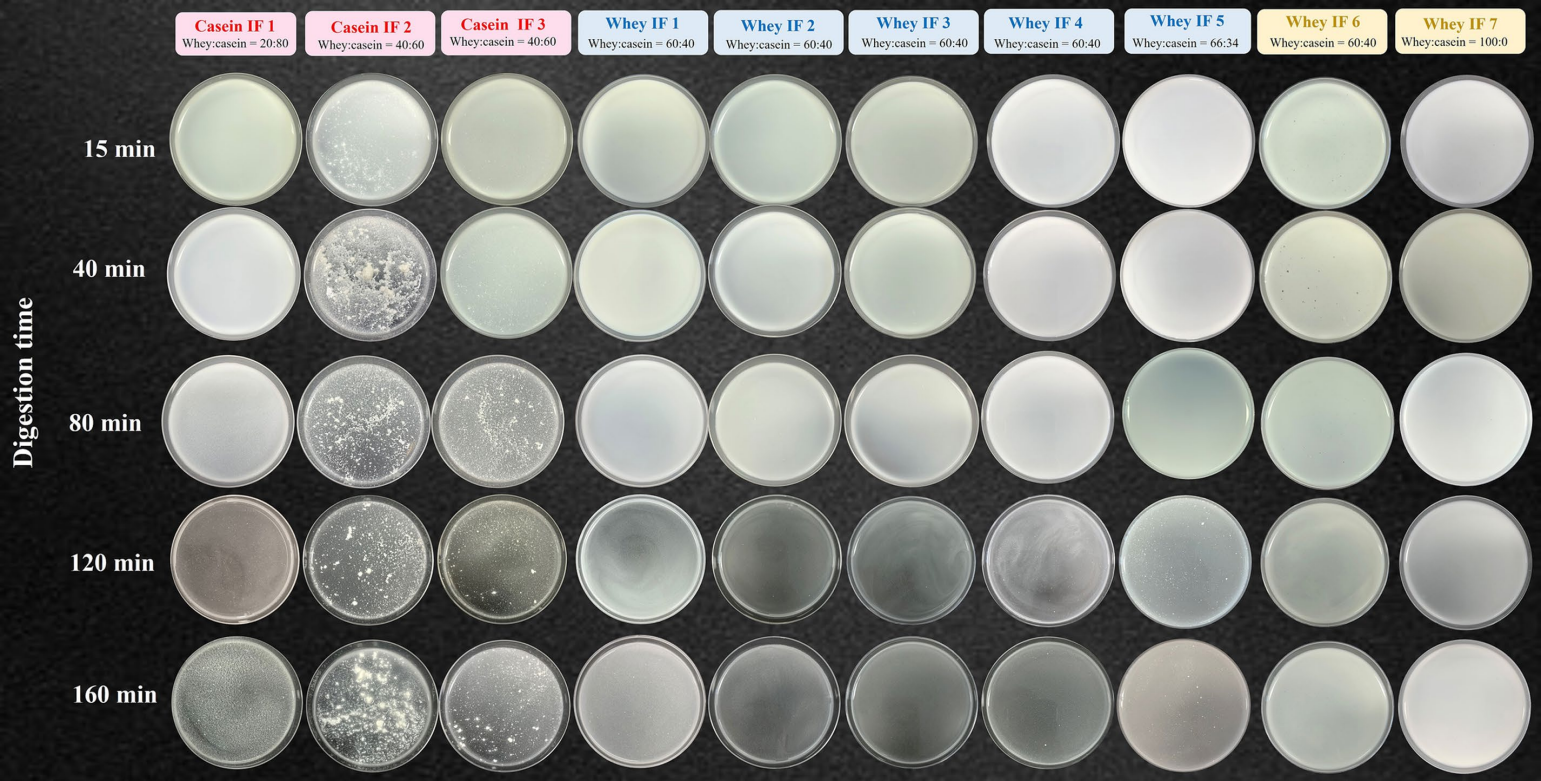
Photographs of gastric chyme of ten commercial infant formulae removed from the iHGS at different digestion times.

The apparent changes in the gastric chyme at different gastric digestion times are shown in Figure 5. After 15 min, casein-dominated IFs 2 and 3 showed early appearance of small curd particles. As digestion time proceeded, the extent of protein coagulation visually increased, as shown by the presence of larger curd particles. Conversely, most whey-dominant infant formulae only exhibited tiny curd particles at the end of digestion, except for whey-dominant biopolymer containing whey IFs 6 and 7 that did not have any visible particles. The formation of curd particles has been attributed to the aggregation of casein micelles, firstly induced by the action of pepsin and then by the gradual acidification of the gastric chyme (23). It is also known that the nature of curd formed is dependent on protein composition and prior heat treatment of milk (36, 44, 45). Therefore it is likely that the differences in the curd formation observed within the casein-dominant IFs were due to different formulations and processing treatments (45–47).

#### 3.2.3 Changes in particle size distribution during digestion

**Figure 6:**
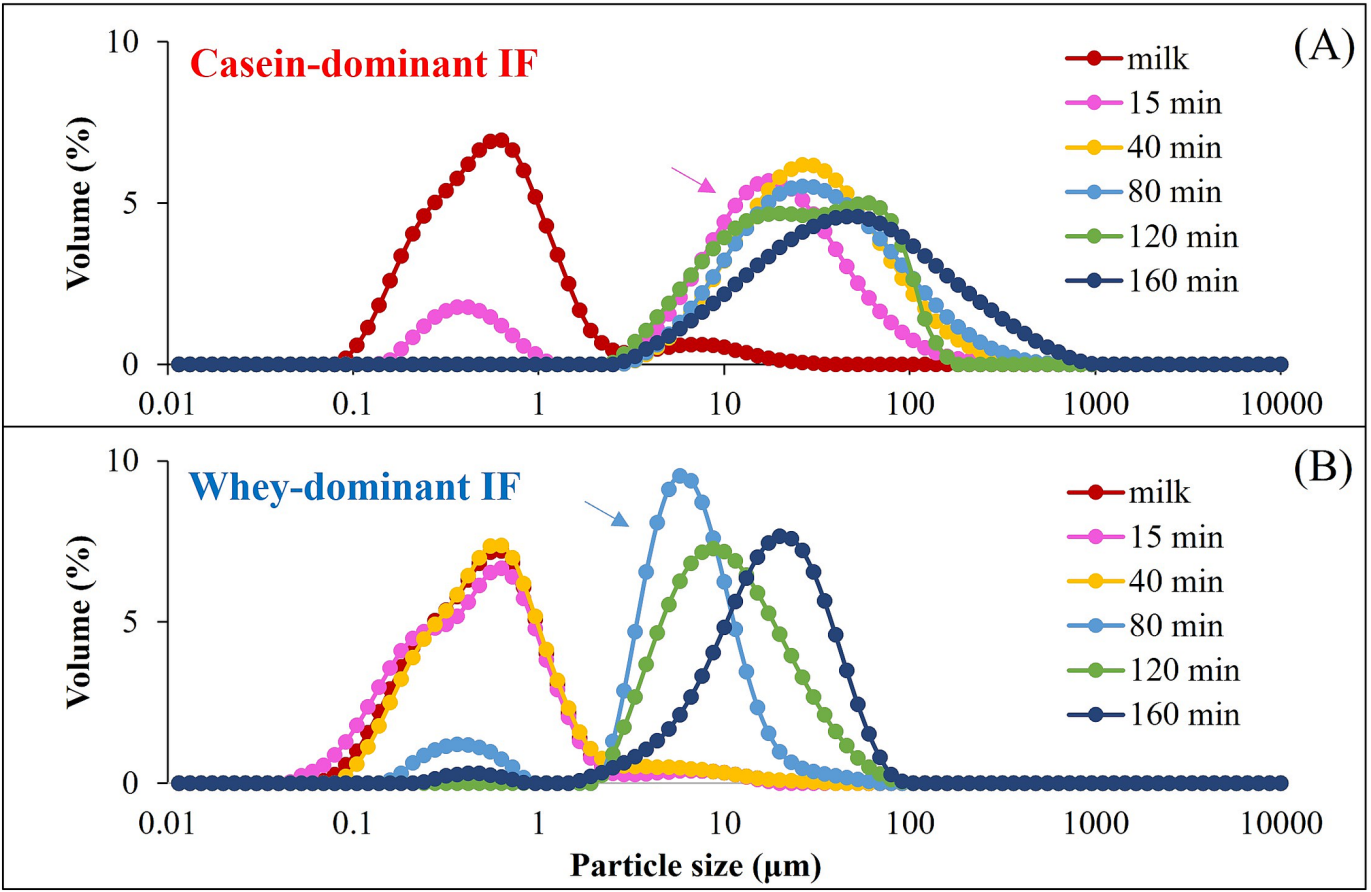
A representative particle size distribution of the casein-dominant IFs (casein IF 2) **(A)** and whey-dominant IFs (whey IF 1) **(B)**. Arrows indicate the onset of aggregation or a shift of the particle size distribution.

A representative particle size distribution of the gastric chyme of a casein-dominant IF (casein IF 2) and of a whey-dominant IF (whey IF 1) at selected digestion times is shown in Figure 6. All the particle size distributions of the gastric chyme of different infant formulae can be found in Supplementary Figures S1, S2. The volume-weighted mean diameters (d_4,3_) can also be found in Supplementary Figure S3. The initial major peak of the undigested IF (0.04–5 μm) gradually shifted towards larger particles from 15 min onwards for casein-dominant IFs, whereas for whey-dominant IFs, this increase in particle size started from 80 min onwards. The increase in particle size of all the infant formulae suggested that the aggregation of milk proteins and/or the flocculation of oil droplets because of the low pH and/or the action of pepsin.

In addition, the differences observed in the onset of aggregation between whey-dominant and casein-dominant groups is in line with our visual observations (section 3.2.2) wherein we observed early signs of aggregation in casein-dominant IFs compared with that of whey-dominant IFs. The differences in the onset and the extent of aggregation between whey-and casein-dominant groups could be driven by protein composition (amount and type of proteins) and interfacial composition of IFs. Previous studies have reported that casein micelles are susceptible to aggregation by pepsin and (or) pH (22), which could be a possible reason for the observed early destabilization and aggregation of casein-dominant IFs in our study. Biopolymer-stabilized whey-dominant IFs (whey IFs 6 and 7) showed a multimodal distribution that could be due to the structured emulsion in the presence of the thickeners.

#### 3.2.4 Microstructural changes during digestion

**Figure 7:**
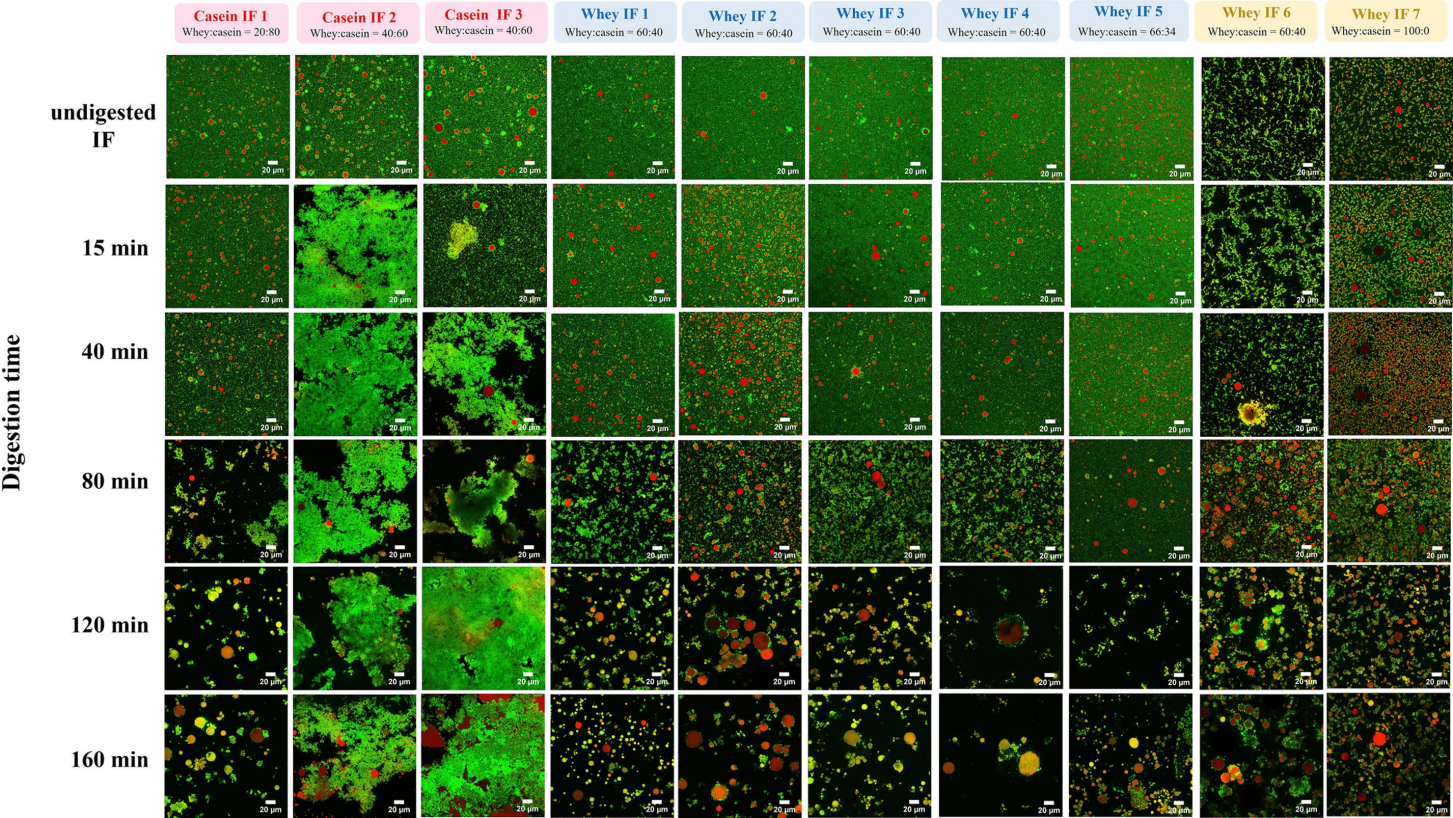
CLSM of gastric chyme from 10 commercial infant formulae at different digestion times. Red color indicates lipid fraction (triacylglycerides) and green color represents protein. Scale bar = 20 μm.

The microstructural arrangements of proteins and lipids in IFs during digestion were further investigated by CLSM as shown in Figure 7. During gastric digestion, casein-dominant formulae showed a higher degree of aggregation and coalescence. Additionally, the extent of protein coagulation within the casein-dominant IF group was greater for casein-dominant goat milk-based infant formulae, followed by casein-dominant sheep-and cow-based infant formulae.

Conversely, whey-dominant formulations showed a lower degree of protein aggregation and greater extent of flocculation of oil droplets. The size (and amount) of the flocs increased, and the embedded oil droplets showed signs of coalescence as the digestion progressed for whey-dominant IFs. Moreover, whey-dominant IFs that contained biopolymers (whey IFs 6 and 7) showed an aggregated structure prior to digestion (undigested IF). This is consistent with the multimodal particle size distribution for the biopolymer-stabilized whey IF 6 and whey IF 7 (Figure 2; Supplementary Figure S2). As digestion progressed, highly flocculated structures with lipid droplets embedded were observed within the biopolymer containing IF group. The high ionic condition in the gastric system promotes colloid aggregation due to the neutralization of the surface charge of the colloid droplets.

The greater degree of coagulation for casein-dominant IFs was related, in general, to the higher proportion of caseins compared with the whey-dominant IFs. However, it is not clear which factors might have influenced the small structural differences in the coagulum formed from cow, goat, and sheep milk during digestion of their respective casein-dominant IFs. A combination of protein composition, pretreatment during processing (such as heating temperatures and homogenization), and other ingredients used during infant formula processing (such as lipid sources, emulsifiers) could influence the structure and properties of protein aggregation during gastric digestion (45, 48). Overall, the microstructural changes observed for different infant formulas were also in line with their visual changes (section 3.2.2) and particle size distribution (section 3.2.3).

#### 3.2.5 Protein hydrolysis

**Figure 8:**
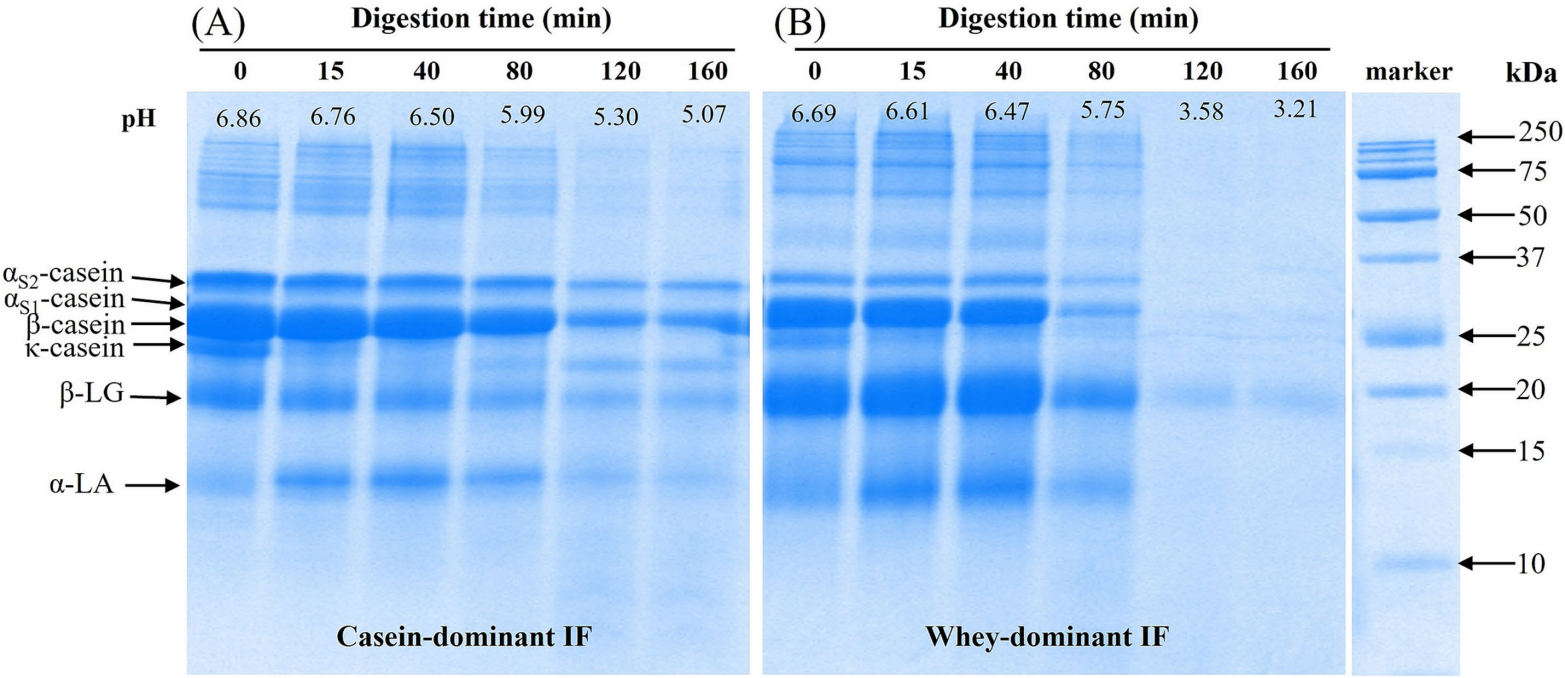
A representative SDS-PAGE under reducing conditions of initial (before digestion) and digested (gastric chyme) of **(A)** casein-dominant Ifs (casein IF 3) and **(B)** whey-dominant IFs (whey IF 2) samples at different time points during digestion in the iHGS. Digestion time at 0 min refers to the undigested IF prior digestion in the iHGS.

A representation of SDS-PAGE results on the protein composition of the gastric chyme of casein-dominant (casein IF 3) and whey-dominant (whey IF 2) IFs during gastric digestion are shown in Figure 8. In all digestion time points, it is evident that casein-dominant IFs (Figure 8A) showed higher proportions of caseins whereas whey-dominant IFs showed higher proportions of whey proteins from *β*-lactoglobulin and *α*-lactalbumin (Figure 8B). After 15 min of gastric digestion, *κ*-casein fraction was found to be hydrolyzed in both formulations. This is due to the high susceptibility of Phe-Met bond of κ-casein to enzyme pepsin resulting in destabilization of casein micelles (49, 50).

The continuous drop of pH and addition of SGF (and enzymes), resulted in further destabilization and coagulation (or aggregation) of casein micelles (as well as casein-whey protein complexes) in all infant formulas, which is in line with previous findings (19, 22, 51). In the casein-dominant IFs, all protein bands slightly decreased in intensity as digestion progressed. This suggested slower hydrolysis of proteins (both caseins and whey proteins), which could be attributed to the extensive aggregation of proteins that limits the access of gastric juices and enzymes in the protein network. The larger aggregates formed in the casein-dominant IFs were influenced not only by the larger fraction of casein but also by the lower proportion of denatured whey protein fraction, which can hinder the aggregation of enzymatically destabilized casein micelles (14, 23).

Contrarywise in whey-dominant IFs, caseins and *α*-lactalbumin bands disappeared and a faint *β*-lactoglobulin band was observed from 120 min digestion. Pepsin enzyme is less constrained in whey-dominant IFs due to the less aggregated structure that results in faster protein (both caseins and whey proteins) hydrolysis during gastric digestion. For both casein-and whey-dominant IFs, a greater extent of hydrolysis was observed for α-lactalbumin compared with β-lactoglobulin. This is expected to be due to the greater susceptibility of α-lactalbumin to pepsin at pH <4 compared with that of β-lactoglobulin which is considered more resistant to hydrolysis by pepsin because of its unique structural stability at low pH (22, 48). β-lactoglobulin is usually influenced by the history of heat-treatment and unfolding and aggregation of the whey proteins (52), however, this information is limited in this study. It must be noted that differences were observed in the pH profiles of casein-and whey-dominant IFs during gastric digestion, which might have also influenced the extent of hydrolysis of proteins. Changes in protein composition of all formulae can be found in Supplementary Figure S4. The extensive protein coagulation/curd formation in casein-dominant infant formulae resulted in retention of caseins in the iHGS for longer times and slowed down the rate of protein hydrolysis (Supplementary Figure S5).

#### 3.2.6 Rate of retention of proteins and lipids in the iHGS

Gastric emptying refers to a complex process in which the stomach discharges its contents into the small intestine to facilitate further digestion and absorption. It is not only related to the mechanical forces and gastro-duodenal pressure gradient in the stomach but also the physicochemical properties of the gastric contents such as structure, particle size, viscosity, caloric density, and volume retained in the stomach (53). To understand the difference in the kinetics of gastric emptying of the infant formulae, the gastric retentions of the key macronutrients (i.e., protein and lipid) were compared.

**Figure 9:**
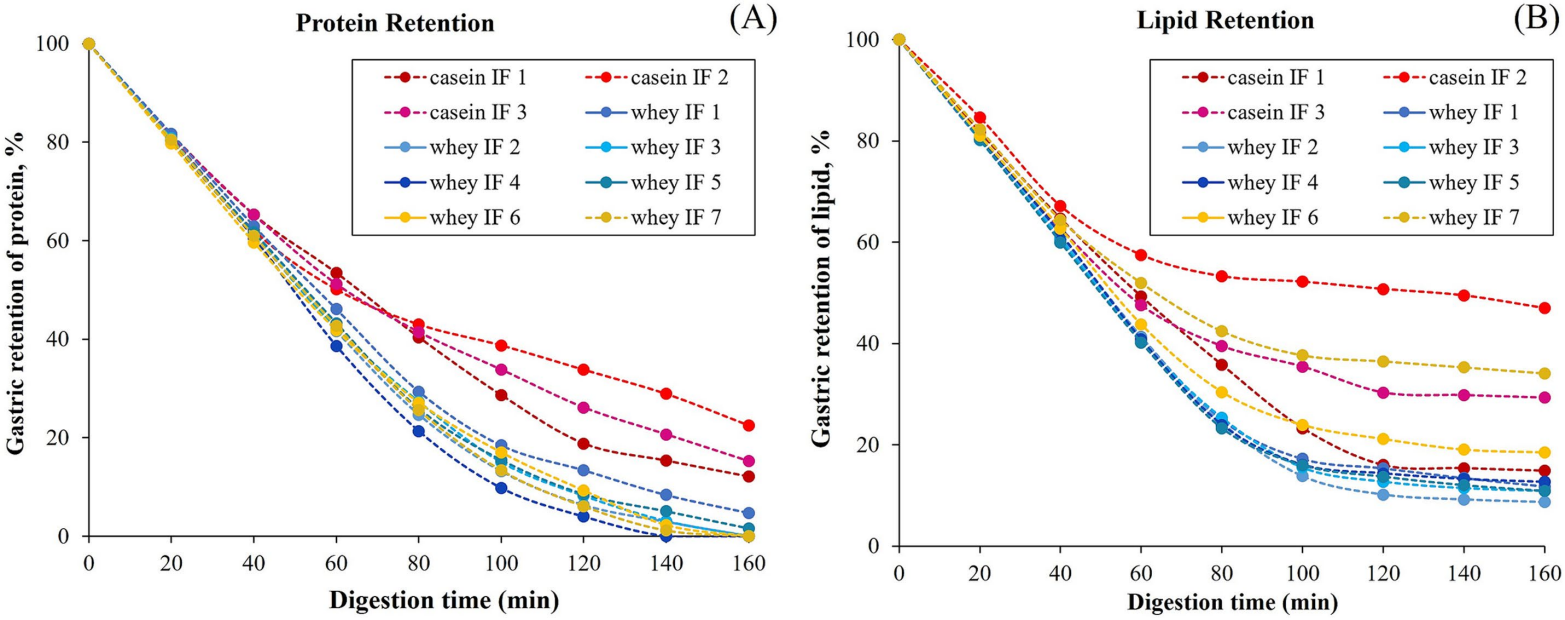
Changes in the protein **(A)** and lipid **(B)** contents retained in the iHGS during digestion.

The changes in the gastric retention of protein and lipid in the iHGS with increasing digestion time are shown in Figures 9A,B, respectively. All the samples displayed a consistent trend of exponential decay of the gastric content with digestion time. Based on the experimental data, the retention of protein was higher for casein-dominant infant formulae compared with the whey-dominant infant formulae as observed from lower rates of emptying, i.e., lower kappa values for casein-dominant IFs (Table 4). This would be related to the large protein aggregates formed in casein-dominant formulae during gastric digestion, as previously discussed (Sections 3.2.2–3.2.4). Similarly, in terms of lipid retention, a higher percentage of retention was found in casein-dominant infant formulae compared with whey-dominant formulae as observed from the kappa values (Table 4).

**Table 4:** Protein and lipid retention kappa values [i.e., the slope of the curve for gastric retention versus digestion time (see Figure 9) calculated from the modified exponential function described in Equation 3].

Sample	Kappa (%/min × 10^−3^)	
	Protein retention	Lipid retention
Casein IF 1	12.0	12.8
Casein IF 2	9.9	6.3
Casein IF 3	11.1	10.2
Whey IF 1	14.6	14.8
Whey IF 2	16.6	15.8
Whey IF 3	16.2	15.4
Whey IF 4	17.7	15.0
Whey IF 5	16.0	15.4
Whey IF 6	16.2	12.9
Whey IF 7	16.6	9.0

The higher retention of lipid in casein-dominant IFs could be due to the lipid droplets being entrapped in the coagulated (or aggregated) curd particles as observed from the microstructure of casein-dominant IFs (confocal scanning laser micrographs). This would have further influenced the rates of lipid emptying from casein-dominant infant formulas as the release of lipids from the casein matrix will be dependent on the breakdown of the surrounding casein network (46). Interestingly biopolymer-stabilized IFs showed higher percentages of lipid retention than the rest of the whey-dominant IFs, which suggests that the presence of thickeners have influenced the lipid delivery. The increased viscosity caused by the thickeners in the formulation slows down the diffusion of lipase accessing the lipid droplets, thereby delaying the lipid delivery.

### 3.3 Analyses on the gastric physical changes and emptying of macronutrients

#### 3.3.1 Correlation between aggregation index, coalescence index, and gastric emptying of proteins and lipids

There are several meal-related and physiological factors that affect gastric emptying and although most meal-related factors can be studied with *in vitro* dynamic gastric digestion models (such as the iHGS), the physiological-related factors cannot be mimicked. In this study, gastric emptying rate is linked to the physical state of the chyme where the degree of intragastric instability of the infant formulae tested relates to the rate of gastric emptying. It is known that food particles larger than 1–2 mm size cannot pass through the pyloric valve of the stomach and move to the small intestine, thus they are pushed back to the stomach by a retropulsion mechanism for further grinding and hydrolysis (30, 53, 54). Therefore, knowing the changes of the food particles formed under gastric conditions is especially important to understand the rate of delivery of nutrients to the duodenum.

**Figure 10:**
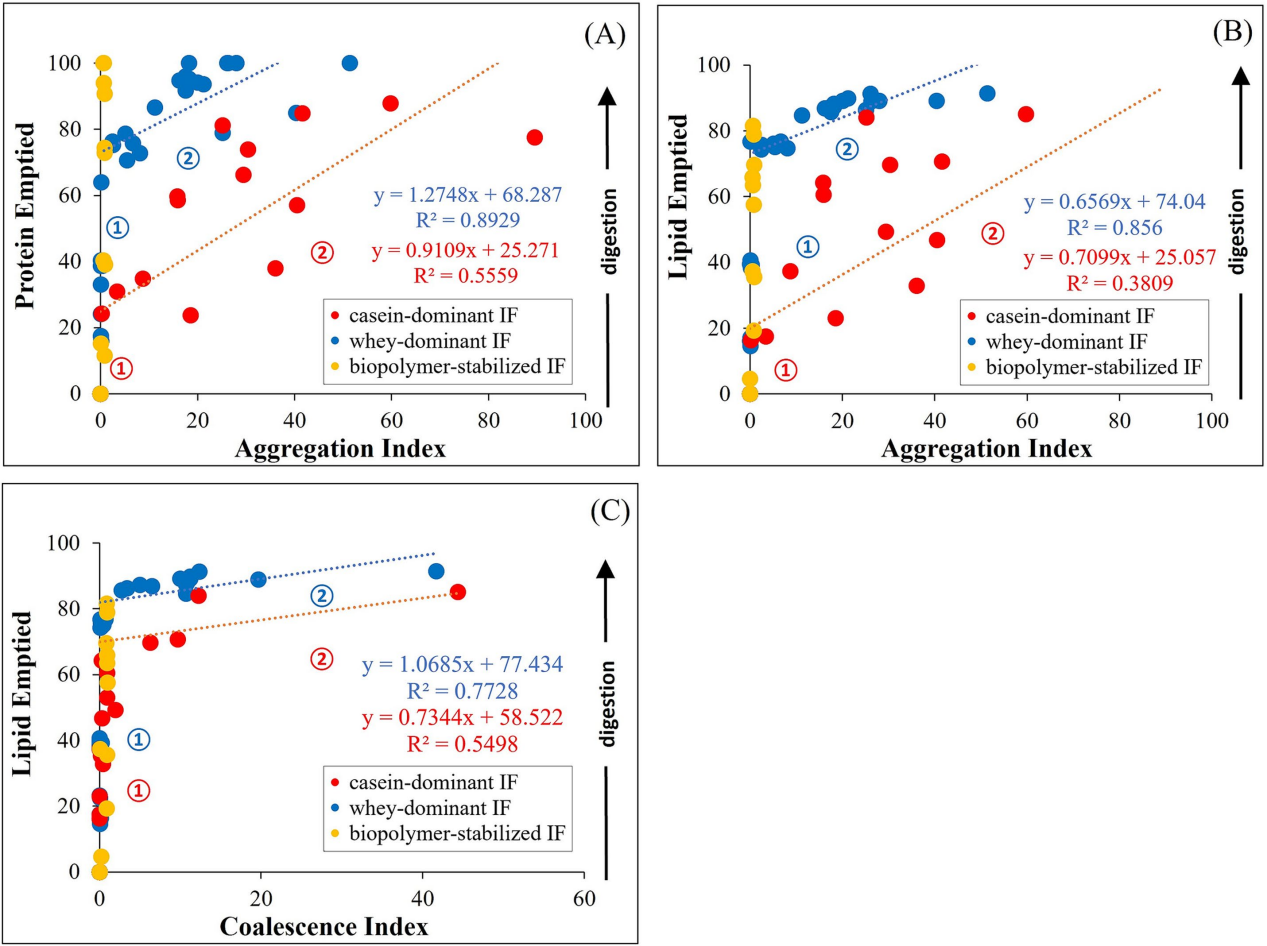
Correlation between aggregation index (AI) and protein **(A)** or lipid **(B)** emptied from the iHGS; and coalescence index (CI) with lipid emptied **(C)**. Numbers indicate the stages in the gastric emptying.

The structural changes and macronutrient delivery discussed previously indicate interrelated behaviors during gastric digestion irrespective of the formulations and processing treatments of the infant formulas. Thus, we further analyzed the correlation of the degree of aggregation (AI) and coalescence (CI) with the macronutrient emptying of proteins and lipids. During gastric digestion, the proportions of protein and lipid emptied correlated with the AI as shown in Figure 10. At early digestion time (stage 1), proteins emptied faster in whey-dominant infant formulae with almost no visible aggregation (Figure 10A).

At a certain digestion time where ~68% proteins (intercept of the fitting line) are emptied (stage 2), AI steadily increased correlated to an almost linear function. This increase in the degree of aggregation relates to a gradual release of protein from the iHGS until most of the proteins are emptied after 3 h of gastric digestion, reaching a projected AI of ~40. In other samples, small degree of flocculated proteins was observed after 160 min digestion which could be attributed to the deviated data points from the projected linear fit in the Figure 10A. Conversely, casein-dominant formulations showed an early onset of increase of AI (stage 1). At ~25% protein emptied AI gradually increased (stage 2), which correlated with a slow protein release during gastric digestion, reaching ~80% protein emptied after 3 h of digestion. The remaining 20% could be attributed to the curd particles retained in the iHGS at the end of the digestion.

In a similar way, lipids also emptied faster in whey-dominant formulae in stage 1 (Figure 10B). After ~74% emptied lipid (Figure 10B), a slower release of lipid was observed, which can be related to a slight increase in the degree of coalescence (CI) as shown in Figure 10C. After the 160 min digestion, ~90% of lipids are emptied, which suggests that ~10% lipid remained in the iHGS that showed slight coalescence (Figures 10B,C). This result could also be supported from the gastric stability images shown in Figure 10 where some of the whey-dominant infant formulae showed creaming in the later digestion times.

On the contrary, casein-dominant infant formulae also showed an early onset of coalescence which resulted in the slower emptying of lipid. The remaining ~75% of lipid showed higher extent of coalescence of which ~25% remained after the end of the digestion. This could be attributed to the dynamic changes in the oil droplets entrapped within the curd structure as seen in the confocal laser scanning micrographs (Figure 7) as observed in previous studies (46, 55).

Whey IFs 6 and 7 were grouped into biopolymer-stabilized IF, which did not show any significant correlation of AI and CI with gastric emptying. This could be attributed to the influence of large particle size of the initial sample emulsion as Equations 1, 2 were calculated normalizing the initial particle size values. This limits the approach of the correlation of degree of aggregation and coalescence in this study. The correlation of the effect of biopolymers to the gastric emptying of infant formulae is still unknown and will be explored in the future.

The kinetics of gastric emptying of proteins and lipids can be deduced into the different stages in gastric environment (stage numbers indicated in Figure 10). Stage 1 can be interpreted as driven by the continuous emptying of proteins and lipid during gastric digestion. The duration of this delivery is influenced by the gastric instability of infant formulae. Stage 1 of casein-dominant IFs appears to be shorter compared with whey-dominant IFs as digestion proceeds due to the early extent of aggregation and coalescence. Stage 2 marks the onset of the destabilization influenced by the infant formulae composition, drop in pH and/or action of pepsin at a certain digestion time. Thus, the kinetics of gastric delivery are affected by protein aggregation and interactions of droplets which results in the formation of flocs or coalesced droplets. Casein-dominant IFs showed an early onset of Stage 2 due to extensive coagulation as described above.

This overall analysis on gastric-induced physical and structural changes and macronutrient protein and lipid emptying generated an essential strategy on the formulation of infant formulae with targeted benefits. The correlated lower degree of aggregation and coalescence to a fast protein hydrolysis and macronutrient delivery in whey-dominant IFs could be relevant to understanding nutrient absorption and availability for infants and the formulation of IFs for specific needs such as for sensitive digestive systems. Similarly, the correlated higher degree of aggregation and coalescence in the casein-dominant IFs which correlated to a slow delivery of macronutrients to the small intestine may possibly be significant to the design of IFs for “hungry babies” and promote better satiation (51). Lastly, the biopolymer-stablized IFs altered the rate of lipid emptying (Figure 9) which could be relevant to formulating IFs to address the gastrointestinal issues of infants such as reflux, colic, and constipation (7–9).

#### 3.3.2 Correlation between gastric protein emptying and lipid emptying

The protein and lipid retention data, as shown in Figure 9, indicated that the changes in the protein and lipid emptied during gastric digestion may be related. The changes in the retention curves (Figure 9) of whey-dominant IFs (except biopolymer-stabilized whey-dominant IFs) show fast protein and lipid retention profiles. Similarly, casein-dominant IFs also show an opposite trend, i.e., slow protein and retention rates.

**Figure 11:**
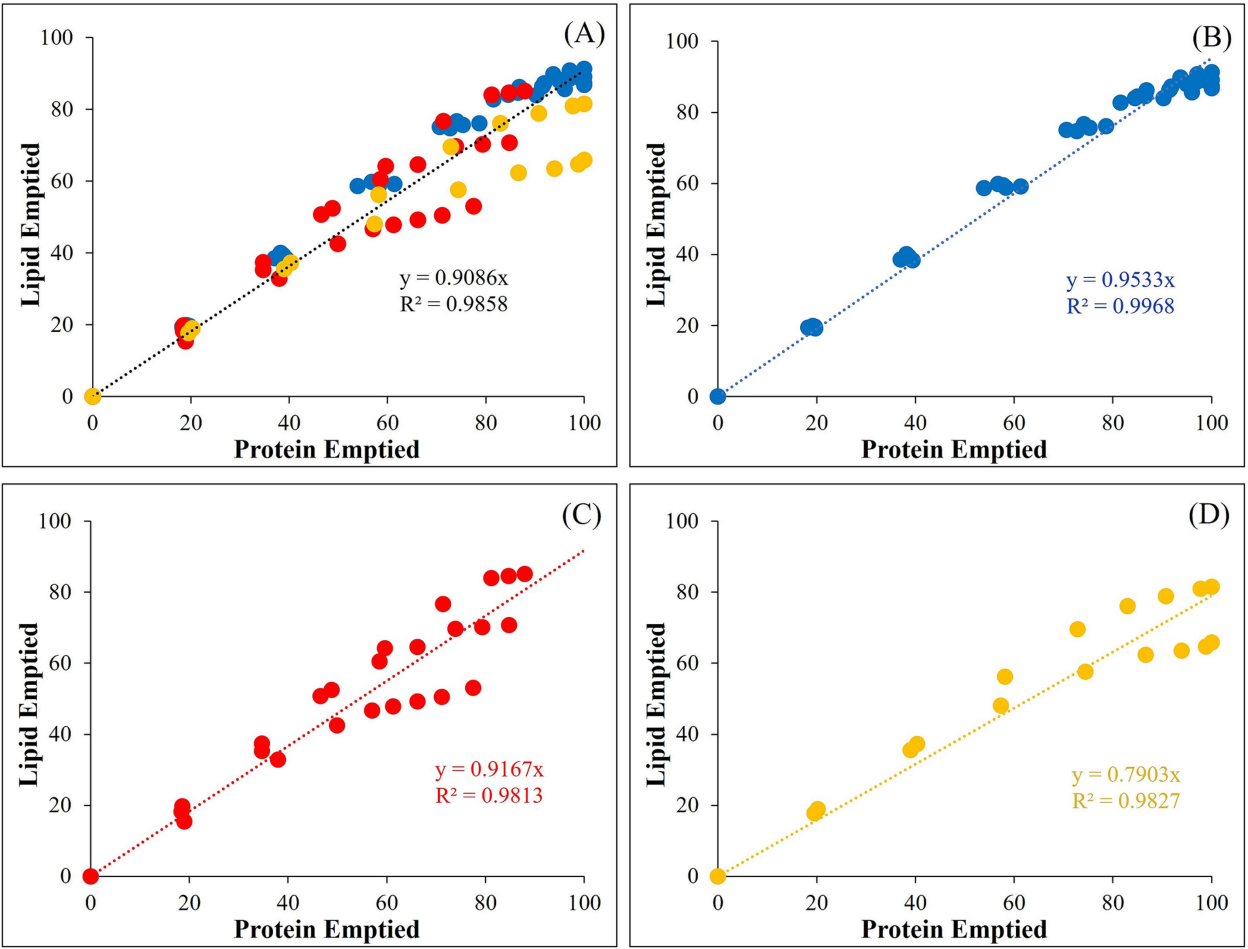
Relationship between the lipid and protein emptied of the different infant formulae during gastric digestion (0–160 min) in the iHGS: **(A)** all of the infant formulae; **(B)** whey-dominant infant formulae (blue); **(C)** casein-dominant infant formulae (red); **(D)** biopolymer-stabilized whey-dominant infant formulae (yellow).

Figure 11 shows the interrelationship between protein and lipid emptied progressively during gastric digestion in the iHGS. For all samples, the linear regression fitting with a slope close to 1.0 (slope = 0.909, R^2^ = 0.986) suggest that protein and lipid emptied have a strong correlation irrespective of the formulation ingredients and processing treatments of the commercial products (Figure 11A). When the data were plotted separately, whey-dominant IFs, casein-dominant IFs, and biopolymer-stabilized IFs showed linear regression fitting slopes of 0.953, 0.917, and 0.790, respectively (Figures 11B–D). Coefficient of determination (R^2^) was between 0.997 and 0.981, which shows a good linear agreement. The linear slope of close to 1.0 suggests that the release of protein is directly proportional to the release of lipid fraction. The lower slope of casein-dominant IFs suggests that lipid was released at a slightly lower rate than protein. This was also confirmed from the lower slope of lipid emptied with AI (Figure 10B). Moreover, the biopolymer-stabilized IFs showed a lower direct proportionality. This could be attributed to the fast protein emptying and slower lipid emptying kinetics as shown in the previous results (Figure 9).

## 4 Conclusion

The *in vitro* gastric digestion behavior of different commercial infant formulae was investigated. The findings of this study underlined the comparable behavior of the infant formulae between whey and casein-dominant formulations as viewed on the correlation of aggregation and coalescence indices to the macronutrient delivery. In casein-dominant IFs, the extensive protein aggregation slowed down the emptying rates of both protein and lipid. CLSM also revealed that oil droplets were entrapped in the curd particles of casein-dominant infant formulae which slowed down the rate of hydrolysis and resulted in retention of caseins in the iHGS for longer times. Conversely, whey-dominant formulations showed lower degree of aggregation and coalescence due to higher proportion of whey proteins in the formulation. Due to the less aggregated structure during gastric digestion, pepsin is less constrained, hence resulting in faster hydrolysis and further causing rapid protein and lipid emptying from the iHGS. It was also revealed that whey-dominant infant formulae in the presence of biopolymers increased the viscosity of gastric chyme and induced droplet flocculation. This change altered the rate of protein hydrolysis and emptying of lipids. The pH profile showed that casein-dominant infant formulae have higher buffering capacity than the whey-dominant infant formulae towards the end of the digestion in the iHGS. This study revealed the effect of formulation composition, proteins, and thickeners, irrespective of the processing background of infant formulae on the digestion properties which may be important for the design of formulae with targeted health benefits.

## Data Availability

The original contributions presented in the study are included in the article/Supplementary material, further inquiries can be directed to the corresponding author.
