# Supplementary material for: Investigation of the gastric digestion behavior of commercial infant formulae using an in vitro dynamic infant digestion model
Source: Front Nutr. 2024 Dec 5;11:1507093. doi: 10.3389/fnut.2024.1507093 (PMC11655231; doi:10.3389/fnut.2024.1507093)
Supplement: Supplementary file 1 [file Data_Sheet_1.docx]

# Supplementary material

**Table S1**. Infant formulae product information based on the product label.

| **Sample** | **Information** |
| --- | --- |
| **Casein IF 1​** | Milk solids, vegetable oils (including soybean), emulsifier (soy lecithin), l-cysteine, choline chloride, l-tryptophan, taurine, inositol, **minerals** (potassium, chloride, calcium, sodium, phosphorus, magnesium, iron, zinc, copper, manganese, iodine, selenium), **vitamins** (Vitamins A, B_1_, B_2_, B_6_, B_12_, C, D, E, K; biotin, folate, niacin, pantothenic acid), **nucleotides** (cytidine 5'-monophosphate, disodium uridine 5'-monophosphate, adenosine 5'-monophosphate, disodium inosine 5'-monophosphate, disodium guanosine 5'-monophosphate), l-carnitine, antioxidant (mixed tocopherols). |
| **Casein IF 2​** | Goat milk, lactose (from milk), vegetable oils (antioxidant (sunflower lecithin, mixed tocopherols)), short chain galacto-oligosaccharides (from milk), dried omega LCPUFAs [contains fish oil, antioxidant (sodium ascorbate, mixed tocopherols, ascorbyl palmitate], long chain fructo-oligosaccharides, choline chloride, l-tyrosine, l-cystine, l-tryptophan, l-isoleucine, taurine, l-carnitine, **minerals** (calcium, sodium, iron, potassium, zinc, copper, manganese, iodine, selenium), **vitamins** (vitamins A, B_1_, B_2_, B_3_, B_5_, B_6_, B_12_, C, D_3_, E, K_1_; folic acid, biotin). |
| **Casein IF 3​** | Lactose, sheep milk solids, vegetable oils [contains emulsifier (soy lecithin), antioxidant (ascorbyl palmitate)], short chain galacto-oligosaccharides, dried omega LCPUFAs [contains fish oil, sodium caseinate, antioxidant (sodium ascorbate, mixed tocopherols, ascorbyl palmitate, dl-α-tocopherol), emulsifier (soy lecithin)], long chain fructo-oligosaccharides, l-cysteine, choline chloride, taurine, inositol, l-carnitine, **minerals** (potassium, calcium, chloride, phosphorus, sodium, magnesium, iron, zinc, copper, iodine, manganese, selenium), **vitamins** (vitamins A, B_1_, B_2_, B_3_, B_5_, B_6_, B_12_, C, D_3_, E, K_1_; folic acid, biotin). |
| **Whey IF 1​** | Milk solids (demineralized whey powder, skim milk, lactose, whey protein concentrate), vegetable oils (contains soy oil), reduced fat cream powder, arachidonic acid (from *M*. *Alpina* oil), fructo-oligosaccharides, docosahexaenoic acid (from *C. Cohnii* oil), emulsifier (soy lecithin), acidity regulators (citric acid, calcium hydroxide, potassium hydroxide), *Bifidobacterium lactis* (DR10™), taurine, inositol, **minerals** (calcium, copper, iron, manganese, magnesium, potassium, iodine, sodium, selenium, zinc), **vitamins** (vitamins A, B1, B2, B3, B5, B6, B12, C, E, D3, K1; β-carotene, biotin, folic acid), **nucleotides** (adenosine 5'-monophosphate, cytidine 5'-monophosphate, guanosine 5'-monophosphate, uridine 5'-monophosphate, inosine 5'-monophosphate). |
| **Whey IF 2​** | Milk solids, vegetable oils, emulsifier (soy lecithin), antioxidant (ascorbyl palmitate), short chain galacto-oligosaccharides (milk), dried omega LCPUFAs [contains fish oil, antioxidant (sodium ascorbate, mixed tocopherols, ascorbyl palmitate], long chain fructo-oligosaccharides, taurine, choline, inositol, l-carnitine, **minerals** (potassium, calcium, chloride, phosphorus, sodium, magnesium, iron, zinc, copper, iodine, manganese, selenium), **vitamins** (vitamins A, B_1_, B_2_, B_3_, B_5_, B_6_, B_12_, C, D, E, K; folate, biotin). |
| **Whey IF 3​** | Milk solids (lactose, demineralized whey powder, whole milk, whey protein concentrate, skim milk), vegetable oils [high oleic sunflower, soy, coconut , canola, emulsifier (soy lecithin), antioxidant (mixed tocopherol)], galacto-oligosaccharide (GOS), dried omega-3 and omega-6 oils [fish oil (tuna), arachidonic acid oil, sodium caseinate, emulsifier (soy lecithin)], acidity regulator (calcium hydroxide, citric acid), choline, taurine, inositol, l-carnitine, **minerals** (potassium, calcium, chloride, phosphorus, sodium, magnesium, iron, Zinc, copper, iodine, manganese, selenium), **vitamins** (vitamins A, B_1_, B_2_, B_3_, B_5_, B_6_, B_12_, C, D, E, K; biotin, folic acid), **nucleotides** (adenosine 5'-monophosphate, cytidine 5'-monophosphate, guanosine 5'-monophosphate, inosine 5'-monophosphate, uridine 5'-monophosphate). |
| **Whey IF 4​** | Raw bovine milk, demineralized whey powder, lactose, 1,3-dioleoyl-2-palmitoylglycerol, soybean oil, anhydrous milk fat, whey protein powder, galactose oligosaccharides, polyfructose, fructose oligosaccharides, whey protein concentrate, phospholipids, arachidonic acid oil (ARA), docosahexa dilute acid oil (DHA), casein phosphopeptide, citric acid, choline chloride, lutein, taurine, l-carnitine tartrate, inositol, calcium carbonate, sodium chloride, ferrous sulfate, zinc sulfate, potassium iodide, copper sulfate, magnesium sulfate, manganese sulfate, potassium chloride, sodium selenite, **minerals** (potassium, calcium, chloride, phosphorus, sodium, iron, zinc, copper, iodine, manganese, selenium), **vitamins** (vitamins A, B_1_, B_2_, B_3_, B_5_ B_6_, B_12_, C, D_3_, E, K; folic acid, d-biotin), **nucleotides** (adenosine 5’-monophosphate, disodium 5'-uridylate, disodium 5'-guanylate, disodium 5'-inosinate, disodium 5'-cytidylate). |
| **Whey IF 5** | Raw bovine milk, lactose, 1,3-dioleoyl-2-palmitoylglycerol, galactose oligosaccharides, vegetable oil (sunflower oil, coconut oil, flaxseed oil, walnut oil), whey protein isolate, demineralized whey protein powder, whey protein concentrate, anhydrous cream, phospholipids, choline, sodium citrate, potassium chloride, copper sulfate, magnesium sulfate, ferric pyrophosphate, zinc sulfate, manganese sulfate, calcium citrate, potassium iodide, sodium selenite, inositol, taurine, l-carnitine, docosahexa dilute acid oil (DHA), arachidonic acid oil (ARA), lutein, casein phosphopeptide, **minerals** (sodium, potassium, magnesium, calcium, chloride, phosphorus, iron, zinc, copper, iodine, manganese, selenium), **vitamins** (vitamins A, B_1_, B_2_, B_3_, B_5_ B_6_, B_12_, C, D, E, K; nicotinic acid, folic acid, biotin), **nucleotides** (disodium 5'-cytidylate, disodium 5'-uridylate, adenosine 5’-monophosphate, disodium 5'-guanylate, disodium 5'-inosinate). |
| **Whey IF 6​** | Milk solids, vegetable oils [contains emulsifier (soy lecithin), antioxidant (ascorbyl palmitate)], thickener (carob bean gum), short chain galacto-oligosaccharides, dried omega LCPUFAs [contains fish oil, antioxidant (sodium ascorbate, mixed tocopherols, ascorbyl palmitate, dl-alpha tocopherol), sodium caseinate, whey protein, emulsifier (soy lecithin)], long chain fructo-oligosaccharides, taurine, choline chloride, inositol, l-carnitine, **minerals** (potassium, calcium, chloride, phosphorus, sodium, magnesium, iron, zinc, copper, iodine, manganese, selenium), **vitamins** (vitamins A, B_1_, B_2_, B_3_, B_5_ B_6_, B_12_, C, D_3_, E, K_1_; folic acid, biotin), **nucleotides** (cytidine 5′-monophosphate, uridine 5′-monophosphate, adenosine 5′-monophosphate, inosine 5′-monophosphate, guanosine 5′-monophosphate). |
| **Whey IF 7​** | Hydrolyzed whey protein concentrate (contains milk), vegetable oils [contains antioxidant (citric acid, mixed tocopherols, ascorbyl palmitate)], glucose syrup, starch, galacto-oligosaccharides (contains milk), lactose, maltodextrin, long chain polyfructose, omega LCPUFAs [contains fish, antioxidant (mixed tocopherols, ascorbyl palmitate)], l-tyrosine, choline chloride, taurine, inositol, l-carnitine, emulsifier (soy lecithin), **minerals** (potassium, calcium, chloride, phosphorus, sodium, iron, zinc, copper, iodine, manganese, selenium), **vitamins** (vitamins A, B_1_, B_2_, B_3_, B_5_ B_6_, B_12_, C, D_3_, E, K_1_; folic acid, biotin), **nucleotides** (cytidine 5′-monophosphate, uridine 5′ monophosphate, adenosine 5′-monophosphate, inosine 5′-monophosphate, guanosine 5′-monophosphate). |

***
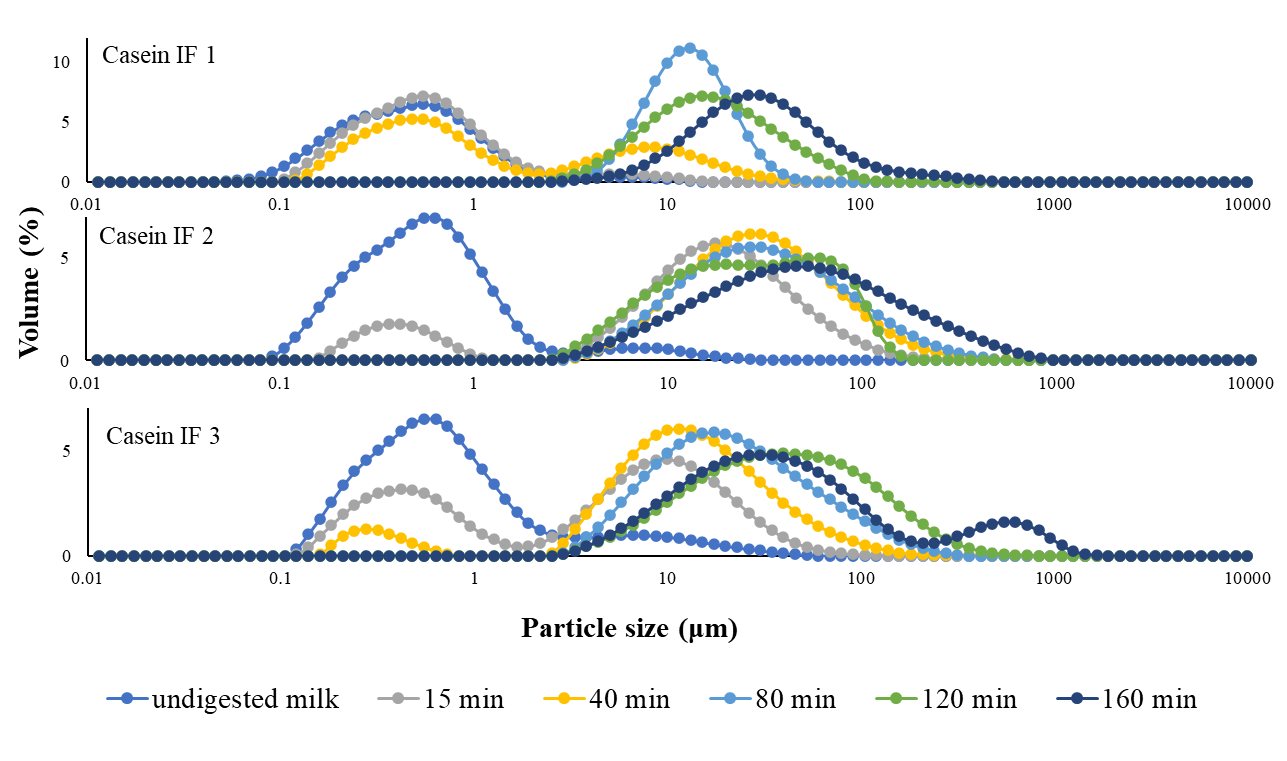
*Figure S1**. Changes in the particle size distribution of the casein-dominant IFs during gastric digestion in the HGS. Casein-dominant IFs 1 and 3 showed onset of aggregation or a shift of the particle size distribution from 40 min, while casein IF 2 showed destabilization from 15 min digestion time.

**
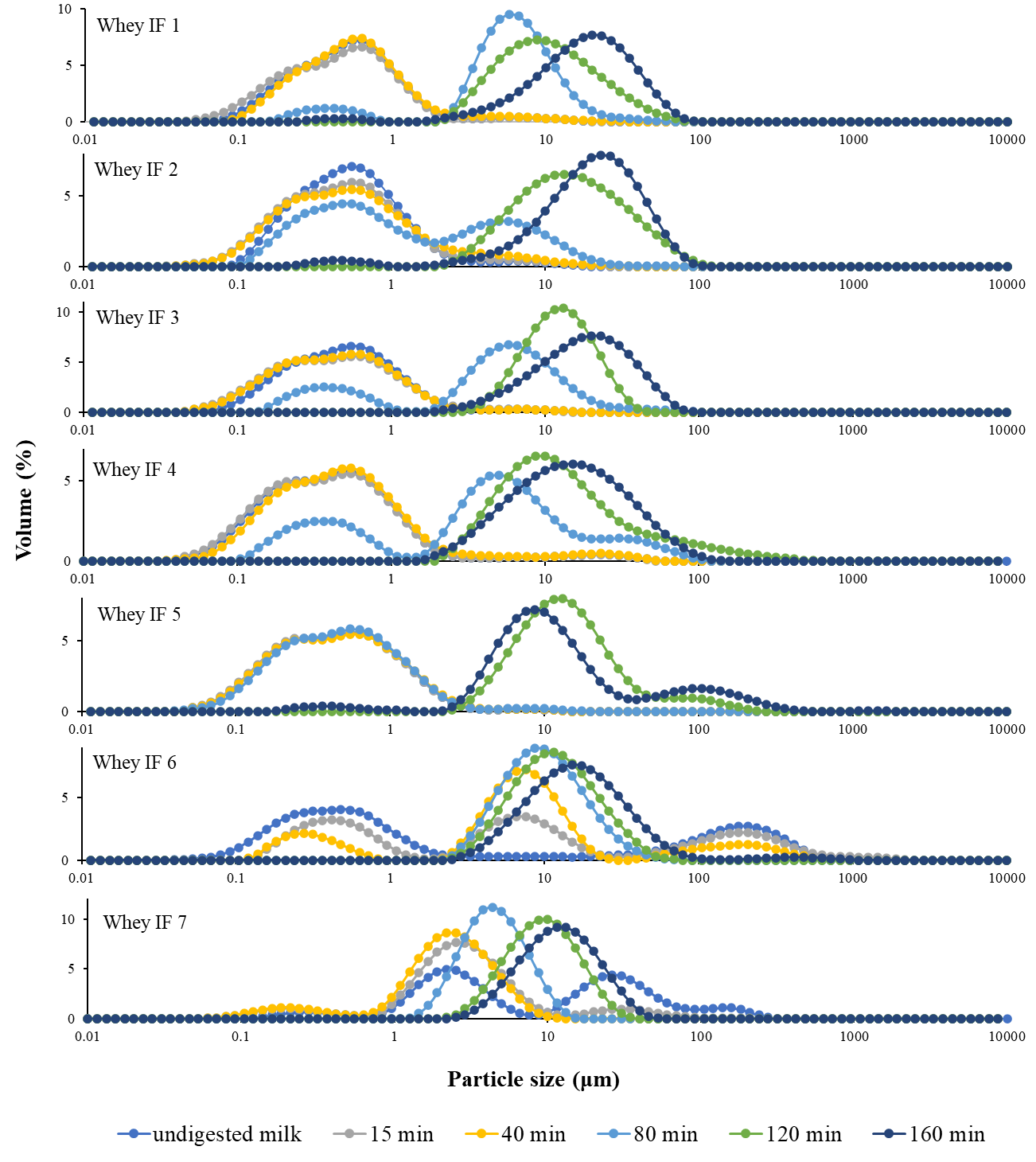
**

**Figure S2**. Changes in the particle size distribution of the whey-dominant IFs during gastric digestion in the HGS. All whey-dominant IFs showed onset of aggregation or a shift of the particle size distribution after 40 min digestion time. Whey IFs 6 and 7 showed multimodal distribution with particles population ranging from 0.04 to 725 µm, which was due to the presence of thickeners (carob bean gum and starch) in the formulation.

***
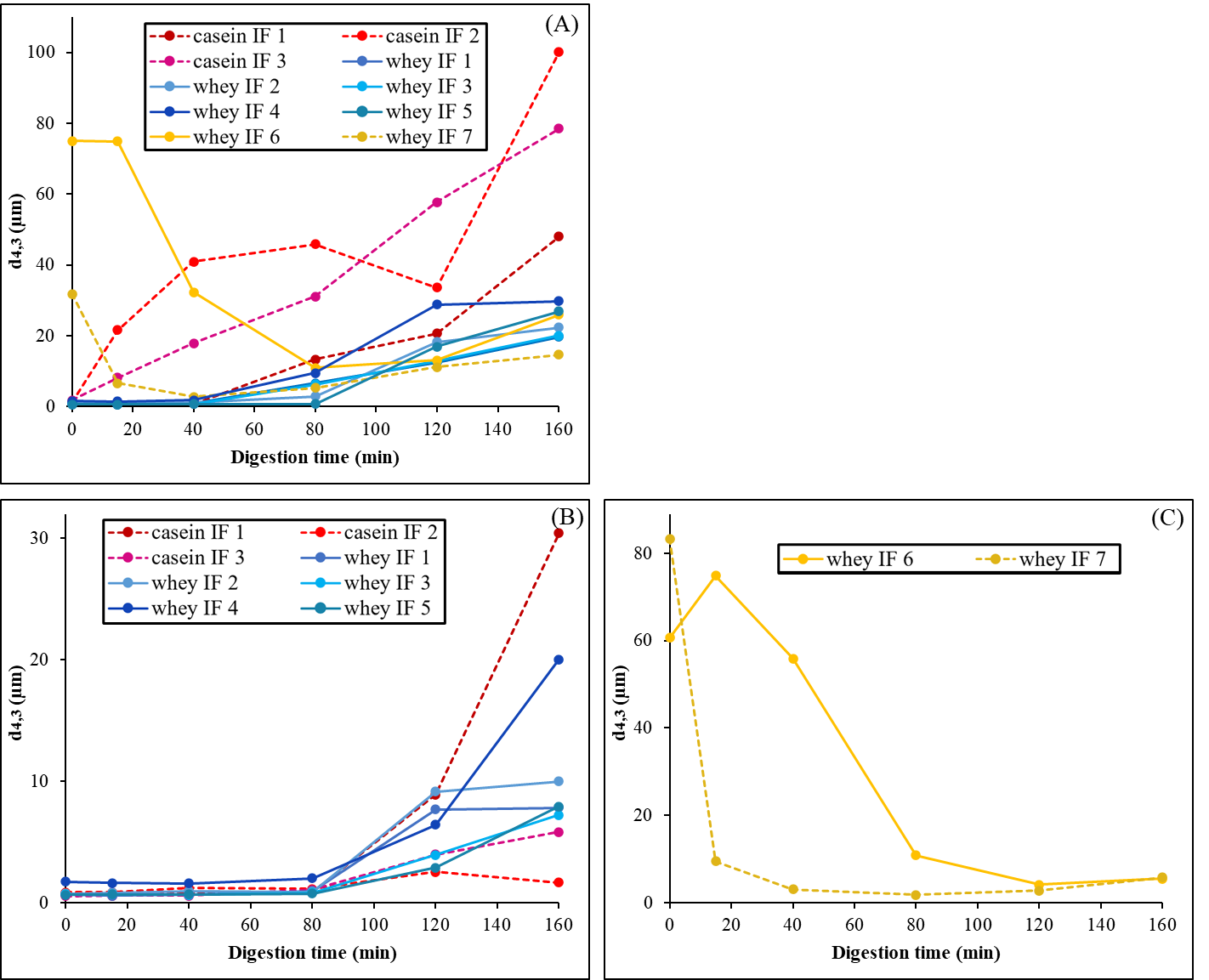
***

**Figure S3**. Changes in the volume-weighted average diameter d_4,3_ of the gastric chyme dispersed in water (A) and SDS+EDTA buffer solution (B) during gastric digestion in the HGS. Data for whey IFs 6 and 7 (C) were removed in (B) as large d_4,3_ values were influenced by the undissolved thickeners. Casein-dominant IFs showed an early increased trend of d_4,3_ values with increased digestion time, which suggested higher extent of aggregation compared with whey-dominant IFs that showed lower extent of destabilization after 40 min.

***
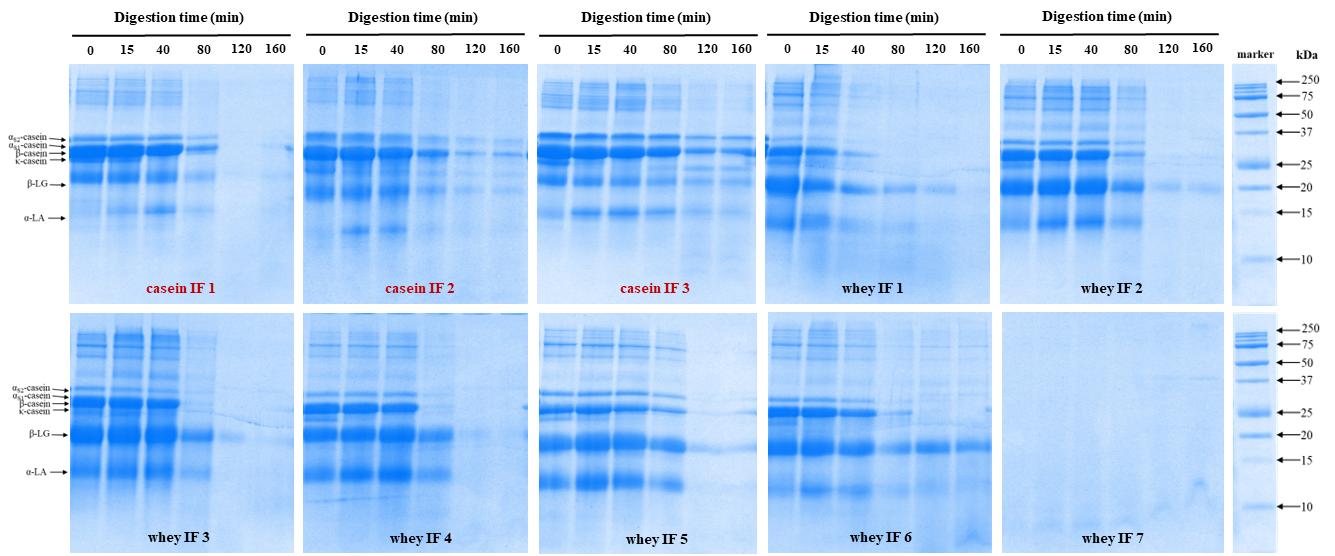
***

**Figure S4**. SDS-PAGE patterns under reducing conditions of initial (before digestion) and digested (gastric chyme) of IFs samples at different time points during digestion in the HGS. Digestion time at 0 min refers to the undigested IF prior digestion in the iHGS. Casein-dominant IFs showed higher casein proportions whereas whey-dominant IFs (except whey IF 7) showed higher proportions of whey proteins from β-lactoglobulin and α-lactalbumin, which confirms the whey to casein ration of the samples. No visible bands were observed with whey IF 7 sample, which confirmed the hydrolyzed proteins in the formulation.


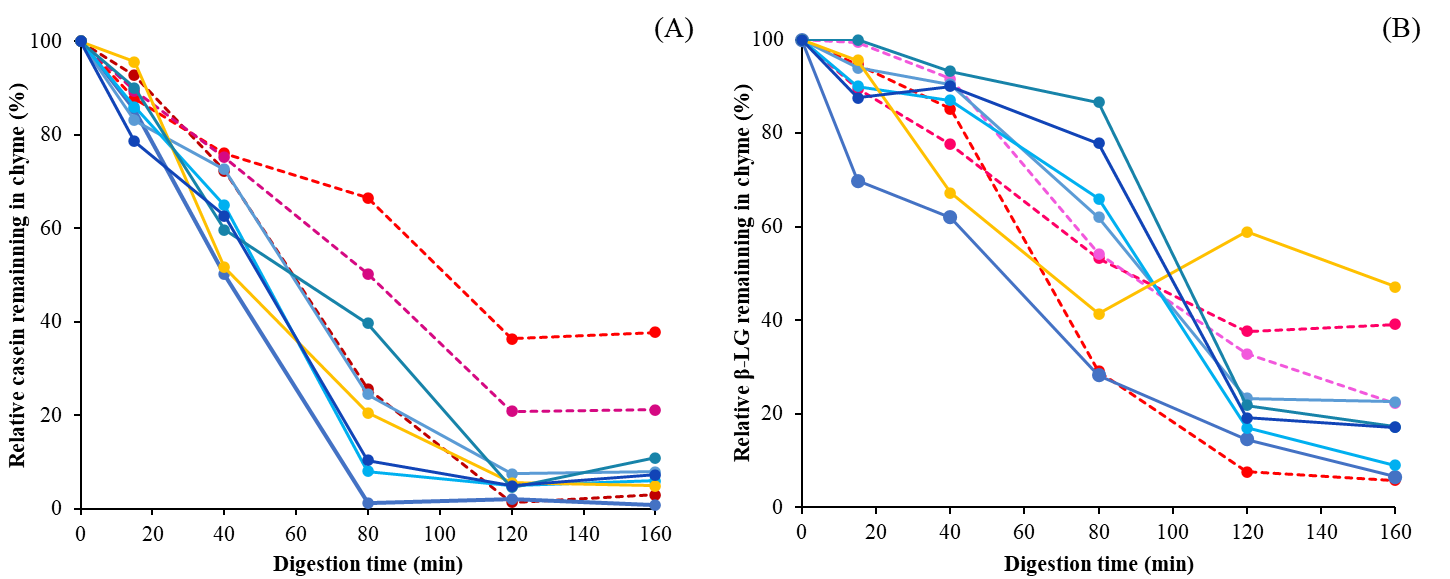


**Figure S5**. Changes in the relative casein (A) and β-lactoglobulin (B) composition during digestion as obtained from SDS-PAGE analysis. Casein-dominant IFs retained higher relative casein in the gastric chyme compared with whey-dominant IFs due to the higher degree of aggregation.
